# Association between triglyceride glucose-waist to height ratio and coronary heart disease: a population-based study

**DOI:** 10.1186/s12944-024-02155-4

**Published:** 2024-06-03

**Authors:** Yangping Zhuang, Yu Wang, Peifen Sun, Jun Ke, Feng Chen

**Affiliations:** 1 Shengli Clinical Medical College of Fujian Medical University, Fujian Medical University, Fuzhou, China; 2 Department of Emergency, Fujian Provincial Hospital, Fuzhou, China; 3 Fujian Provincial Key Laboratory of Emergency Medicine, Fuzhou, China

**Keywords:** TyG-WHtR, Coronary heart disease, NHANES, Adults, Cross-sectional analysis

## Abstract

**Background:**

The Triglyceride glucose (TyG) index-related indicators improve risk stratification by identifying individuals prone to atherosclerosis early in life. This study aimed to examine the relation between TyG-waist circumference-to-height ratio (TyG-WHtR) and coronary heart disease.

**Methods:**

Data from four National Health and Nutrition Examination Surveys (NHANES) cycles between 2011 and 2018 were used for a cross-sectional study. The association between TyG-WHtR and coronary heart disease risk was examined using a multifactorial logistic regression model, and corresponding subgroup analyses were performed. Nonlinear correlations were analyzed using smooth curve fitting and threshold effects analysis. When nonlinear connections were discovered, appropriate inflection points were investigated using recursive methods.

**Results:**

TyG-WHtR and coronary heart disease were significantly positively correlated in the multifactorial logistic regression analysis. Subgroup analyses and interaction tests revealed that gender, age, smoking status, and cancer were not significantly associated with this correlation (***P*** for interaction > 0.05). Furthermore, utilizing threshold effect analysis and smooth curve fitting, a nonlinear connection with an inflection point of 0.36 was observed between TyG-WHtR and coronary heart disease.

**Conclusions:**

According to this study, the American population is far more likely to have coronary heart disease if they have higher TyG-WHtR levels.

**Supplementary Information:**

The online version contains supplementary material available at 10.1186/s12944-024-02155-4.

## Introduction

Cardiovascular disease, one of the conditions with the highest rates of morbidity and mortality globally, presents a public health challenge as well as a significant financial and psychological burden on patients and their families [1]. Approximately one-quarter of all deaths in the US are attributed to coronary heart disease annually [2]. Atherosclerosis and coronary heart disease are closely associated; in coronary heart disease, atherosclerotic plaques form in the coronary arteries that supply the myocardium, reducing blood flow perfusion to the heart [3]. Metabolic syndrome plays a significant role in the development of coronary heart disease, with varying levels of correlation between the incidence and prevalence of coronary heart disease, suggesting that there may be specific common disease progressions between them. Diabetes mellitus, dyslipidemia, and metabolic dysfunction, a collection of concurrent conditions characterized by insulin resistance [4, 5], are of great interest in actively investigating the precise mechanisms by which metabolic dysfunction influences the onset of coronary heart disease. Determining relevant pathways is crucial for diagnosing, treating, and preventing coronary heart disease.

Insulin resistance (IR) is a condition in which the body is less sensitive and responsive to the effects of insulin. Patients with IR are more likely to develop various metabolic diseases, including dyslipidemia, hypertension, and abnormal blood sugar levels. These conditions increase the risk of atherosclerosis, inflammation, and coagulation abnormalities and are also significantly associated with a worse prognosis for cardiovascular disease [1, 6–8]. The TyG index is a correlative indicator of metabolic dysfunction that determines the levels of glucose and triglycerides in fasting blood. The TyG index has recently gained recognition as a reliable and user-friendly alternative to IR with significant therapeutic implications for identifying metabolic dysfunction [9]. The TyG index outperformed the homeostasis model assessment of insulin resistance in predicting coronary artery calcification [10]. It is substantially correlated with carotid atherosclerosis, even after controlling for conventional cardiovascular risk factors [11]. In individuals with type 2 diabetes, Chen et al. recently noted an independent correlation between the TyG index and severe coronary artery stenosis [12]. The TyG index is linked to an increased risk of cardiovascular disease, atherosclerosis, and coronary artery calcification [10, 13]. The TyG-related index is a more reliable predictor of IR and cardiometabolic risk than is the TyG index alone [14, 15]. Additionally, correlative indices, such as Triglyceride glucose-body mass index (TyG-BMI), Triglyceride glucose-waist circumference (TyG-WC), and Triglyceride glucose-waist circumference-to-height ratio (TyG-WHtR), which incorporate anthropometric measurements of obesity in addition to TyG, are more accurate predictors of Insulin resistance and cardiometabolic risk than is the TyG index alone [16–19]. Furthermore, the integrated association score of TyG and anatomical indicators of obesity is a more precise indicator of the degree of insulin resistance. Therefore, early monitoring of insulin resistance in obese individuals is crucial to avoid a poor prognosis in coronary heart disease.

To the best of our knowledge, there is inadequate data on the association between TyG-WHtR levels and coronary heart disease. Identifying practical and helpful markers that allow early intervention in metabolic factors in individuals at high risk of coronary heart disease is crucial. Thus, our investigation aimed to examine the association between TyG-WHtR indices and the risk of coronary heart disease using a large-scale, cross-sectional, population-based study.

## Methods

### Data sources and study population

The Centers for Disease Control and Prevention conducted the NHANES study of the ambulatory population in the United States to evaluate the health, nutritional status, and lifestyle of Americans. The NHANES employs a sophisticated multistage stratified sampling strategy and releases data biennially. The National Center for Health Statistics Research Ethics Review Board approved all technique utilized in the NHANES study. All survey respondents provided written informed consent. During the survey, participants completed several questions and pertinent tests. The five main components of the NHANES database are screening, diet, laboratory, questionnaire, and demographic data. This analysis used data from four NHANES cycles from 2011 to 2018.

**Fig. 1:**
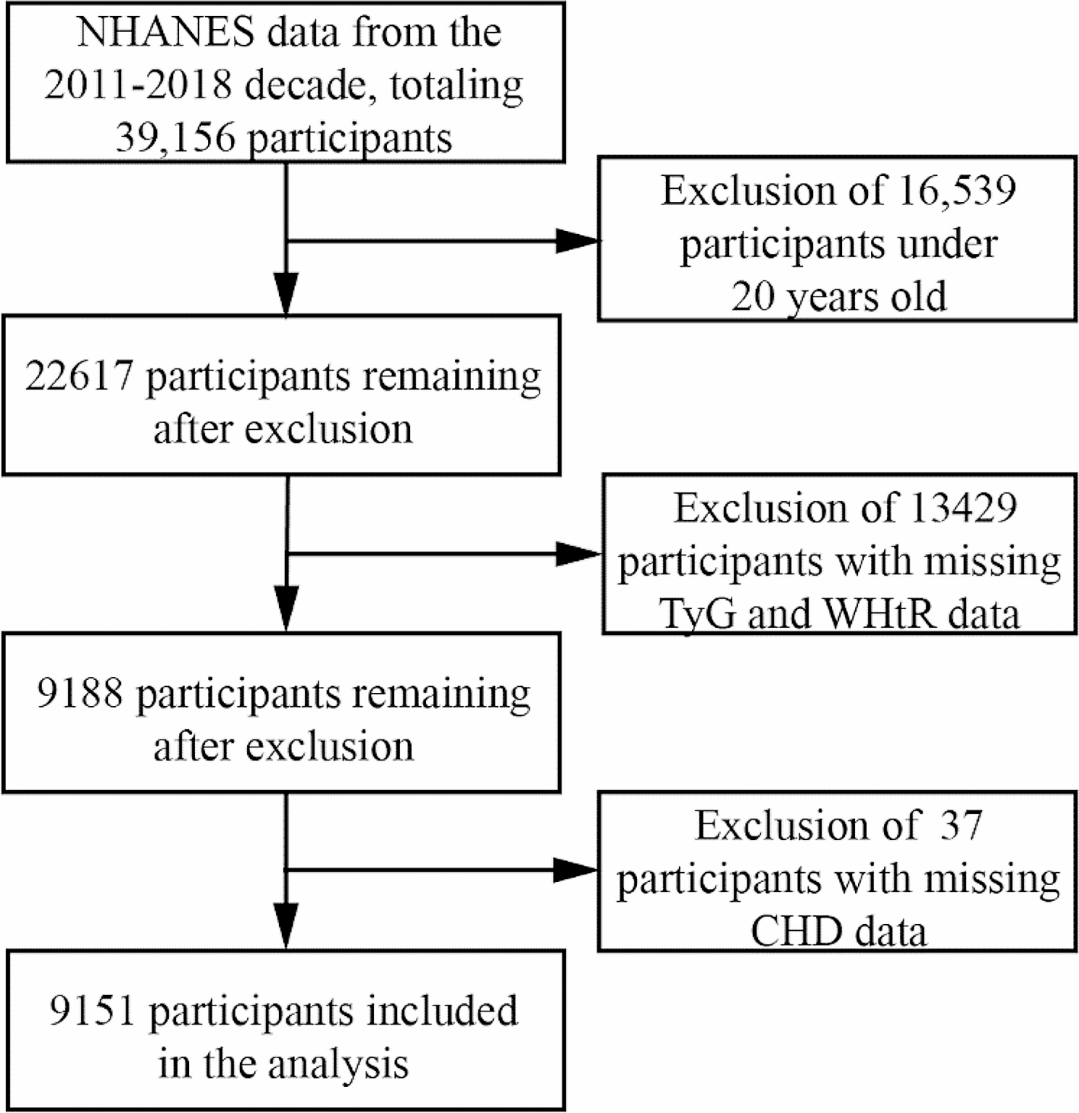
Flow chart

Studies on research designs have been published [20]. Overall, 39,156 participants were identified from the NHANES statistics spanning 2011 to 2018. The exclusion criteria included: 16,539 participants under 20 years of age, 13,429 participants with missing TyG and WHtR data, and 37 participants with missing coronary heart disease data. Ultimately, we analyzed the outcome data of 9,151 participants (Fig. 1).

### Definitions of TyG-WHtR and coronary heart disease

The TyG index measures triglyceride and fasting blood glucose levels to quantify insulin resistance. The following formula was used to calculate the correlation index: TyG = Ln[fasting blood glucose(mg/dL)×fasting triglyceride(mg/dL)/2] [21]; WHtR = waist circumference/height; TyG-WHtR = TyG×WHtR [18]. The exposure variable in our study was TyG-WHtR.

A trained interviewer asked patients a series of questions about their coronary heart disease history. Participants were asked, “Has a doctor or other health professional ever told you that you had coronary heart disease?” They responded with either “yes” or “no.” Those who were unsure or declined to respond were considered absent. Coronary heart disease was the outcome variable in our analysis [22].

### Study variables

The covariates included age, gender, race, annual household income, education level, smoking status, drinking status, hypertension, diabetes, dietary supplement use, regular exercise, cancer, and cholesterol levels. Race was categorized as Mexican American, Other Hispanic, Non-Hispanic White, Non-Hispanic Black, or Other. Educational level was designated as less than 9th grade, 9th–11th grade, high school graduate, AA degree, or college graduate or above. Annual household income was grouped with a threshold of USD 100,000. Smoking status was determined by having smoked more than 100 cigarettes in a lifetime. Drinking status was defined based on consuming more than 12 drinks per year, with those answering “yes” considered drinkers. Medical professionals confirmed diagnoses of hypertension, diabetes, and cancer. Waist circumference and height, vital measurements taken during a medical checkup, along with fasting blood glucose, triglyceride, and total cholesterol levels, were assessed using relevant laboratory tests. For more detailed information on how these variables were measured, please refer to visit the NHANES website: www.cdc.gov/nchs/nhanes/.

### Statistical analysis

All statistical analyses were performed by considering intricate multistage cluster surveys and using proper NHANES sampling weights in compliance with the Centers for Disease Control and Prevention criteria. The participants were divided into groups based on the presence of TyG-WHtR and coronary heart disease. Continuous data are represented as mean ± standard deviation, and categorical variables are expressed as frequency percentages [20]. All variables were evaluated for variance using the chi-square test, nonparametric Kruskal–Wallis test, or analysis of variance. The participants were divided into groups depending on the presence of TyG-WHtR and coronary heart disease. To identify any possible correlation between the TyG-WHtR index (continuous or tertile) and coronary heart disease, multivariate logistic regression analysis was used to determine the odds ratio (OR) and 95% confidence interval (CI) in three distinct models. Using multivariate regression analysis, the independent relation between TyG-WHtR and coronary heart disease was examined using three distinct models. The crude model was not adjusted for correlation, the minimally adjusted model was adjusted for age, gender, and race, and the fully adjusted model was adjusted for education level, smoking status, drinking status, hypertension, diabetes, dietary supplement use, regular exercise, cancer, and cholesterol. The model was fully adjusted for age, gender, and race. The subgroup analyses of TyG-WHtR and coronary artery disease were stratified using criteria based on gender, age, smoking status, hypertension, and cancer. A threshold effect analysis was used to examine the relation and inflection points between TyG-WHtR and coronary heart disease. Finally, we characterized the nonlinear connection between TyG-WHtR and coronary heart disease risk using smooth curve fitting. R Studio (version 4.3.1) and EmpowerStats (version 2.0) were used for statistical analysis. Statistical significance was set at *P*<0.05.

## Results

### Baseline characteristics of participants

A total of 9,151 individuals were included in the study, with a mean age of 49.57 ± 17.38 years. Among the participants, 51.31% were male and 48.69% were female. The racial composition was as follows: 13.51% Mexican Americans, 10.83% Other Hispanics, 37.92% Non-Hispanic White, 21.21% Non-Hispanic Black, and 16.53% from other racial backgrounds. Coronary heart disease was diagnosed in 4.04% of the participants.

The clinical features of the individuals are listed in Table 1, arranged according to the TyG-WHtR tertiles. The tertiles were statistically significant for age, gender, race, annual household income, educational level, smoking status, drinking status, hypertension, diabetes, cancer, cholesterol, and coronary heart disease (*P* < 0.05). Participants in the Tertiles 3 group tended to be older, male, and Non-Hispanic White, with an annual household income under $100,000, a higher education level, and higher cholesterol levels.

**Table 1:** Characteristics of the study population based on TyG-WHtR

TyG-WHtR	Tertiles 1 (*N* = 3050)	Tertiles 2 (*N* = 3050)	Tertiles 3 (*N* = 3051)	*P* value
Age (years)	43.91 ± 17.65	51.01 ± 17.21	53.79 ± 15.71	< 0.001
gender (%)				< 0.001
Male	42.46%	51.11%	52.51%	
Female	57.54%	48.89%	47.49%	
Race (%)				< 0.001
Mexican American	8.82%	13.84%	17.86%	
Other Hispanic	7.90%	11.74%	12.85%	
Non-Hispanic White	35.05%	37.21%	41.49%	
Non-Hispanic Black	29.70%	20.52%	13.41%	
Other Races	18.52%	16.69%	14.39%	
Annual household income (%)				< 0.001
Yes	22.10%	19.16%	13.40%	
No	77.90%	80.84%	86.60%	
Education level (%)				< 0.001
Less than 9th grade	5.35%	9.05%	12.46%	
9-11th grade	11.02%	13.34%	15.25%	
high school graduate AA degree College graduate or above	21.03% 31.30% 31.30%	22.26% 30.23% 25.11%	22.20% 29.68% 20.40%	
Smoking status (%)				< 0.001
Yes	37.16%	44.69%	48.28%	
No	62.84%	55.31%	51.72%	
Drinking status (%)				0.013
Yes	1.48%	1.63%	2.68%	
No	98.52%	98.37%	97.32%	
Hypertension (%)				< 0.001
Yes	24.35%	35.70%	50.56%	
No	75.65%	64.30%	49.44%	
Diabetes (%)				< 0.001
Yes	3.68%	10.44%	28.60%	
No	96.32%	89.56%	71.40%	
Dietary supplements use (%)				0.368
Yes No	51.87% 48.13%	53.05% 46.95%	53.64% 46.36%	
Regular exercise (%)				0.069
Yes No	21.59% 78.41%	19.84% 80.16%	19.31% 80.69%	
Cancer (%)				< 0.001
Yes No	6.82% 93.18%	9.54% 90.46%	10.96% 89.04%	
Cholesterol (mmol/L)	4.55 ± 0.91	4.95 ± 1.03	5.21 ± 1.17	< 0.001
Coronary heart disease (%)				< 0.001
Yes	2.10%	4.30%	5.74%	
No	97.90%	95.70%	94.26%	

Mean ± SD for continuous variables: the ***P*** value was calculated by the weighted linear regression model

(%) For categorical variables, the ***P*** value was calculated using the chi-square test

The clinical features of the individuals are listed in Table 2, according to whether they had coronary heart disease. Age, gender, race, annual household income, educational level, smoking status, hypertension, diabetes, dietary supplement use, regular exercise, cancer, cholesterol level, and the TyG-WHtR were significantly associated with the presence or absence of coronary heart disease (*P* < 0.05). Patients with coronary heart disease tended to be older, male, Non-Hispanic White, with an annual household income under $100,000, educated to an AA degree, with smoking status, hypertension, dietary supplement use, and higher TyG-WHtR levels than those without the condition.

**Table 2:** Characteristics of the study population based on coronary heart disease

	Coronary heart disease (*N* = 370)	Non-coronary heart disease (*N* = 8781)	*P* value
Age (years)	68.55 ± 10.54	48.77 ± 17.15	< 0.001
Gender (%)			< 0.001
Male	64.86%	48.01%	
Female	35.14%	51.99%	
Race (%)			< 0.001
Mexican American	7.57%	13.76%	
Other Hispanic	9.73%	10.88%	
Non-Hispanic White	58.65%	37.05%	
Non-Hispanic Black	14.05%	21.51%	
Other Races	10.00%	16.81%	
Annual household income (%)			0.002
Yes	12.03%	18.48%	
No	87.97%	81.52%	
Education level (%)			0.001
Less than 9th grade	14.59%	8.72%	
9-11th grade	13.51%	13.19%	
high school graduate AA degree College graduate or above	21.35% 30.00% 20.54%	21.85% 30.42% 25.82%	
Smoking status (%)			< 0.001
Yes	59.19%	42.71%	
No	40.81%	57.29%	
Drinking status (%)			0.156
Yes	0.52%	1.95%	
No	99.48%	98.05%	
Hypertension (%)			< 0.001
Yes	77.51%	35.16%	
No	22.49%	64.84%	
Diabetes (%)			< 0.001
Yes	39.15%	13.14%	
No	60.85%	86.86%	
Dietary supplements use (%)			< 0.001
Yes No	68.92% 31.08%	52.18% 47.82%	
Regular exercise (%)			0.036
Yes No	15.95% 84.05%	20.43% 79.57%	
Cancer (%)			< 0.001
Yes No	22.16% 77.84%	8.56% 91.44%	
Cholesterol (mmol/L)	4.41 ± 1.17	4.92 ± 1.06	< 0.001
TyG-WHtR	0.94 ± 0.53	0.73 ± 0.47	< 0.001

Mean ± SD for continuous variables: the ***P*** value was calculated by the weighted linear regression model

(%) For categorical variables, the ***P*** value was calculated using the chi-square test

### Relation between TyG-WHtR and coronary heart disease

The findings of the multivariate regression analysis of TyG-WHtR and coronary heart disease are shown in Table 3. The fully adjusted model revealed that the association between TyG-WHtR and coronary heart disease was significantly positive (OR = 1.83; 95% CI: 1.28–2.62, *P* = 0.0008), indicating a higher TyG-WHtR value increases the odds of developing coronary heart disease. This association was evident in both the crude (OR = 2.26; 95% CI: 1.87–2.72, *P* < 0.0001) and minimally adjusted model (OR = 1.85; 95% CI: 1.48–2.32, *P* < 0.0001). For additional sensitivity analyses, TyG-WHtR was converted from continuous to categorical variables (Tertiles 1, 2, and 3). In the fully adjusted model, participants in Tertile 3 had a 77% higher risk of coronary heart disease than those in Tertile 1 (OR = 1.77; 95% CI: 1.13–2.76, *P* = 0.0121). Furthermore, all three models had trend tests with ***P*** values of < 0.05, indicating statistical significance.

**Table 3:** Association between TyG-WHtR and coronary heart disease

	OR (95%CI), *P*-value		
	Crude model	Minimally adjusted model	Fully adjusted model
TyG-WHtR	2.26 (1.87, 2.72) < 0.0001	1.85 (1.48, 2.32) < 0.0001	1.83 (1.28, 2.62) 0.0008
TyG-WHtR (Tertiles)			
Tertiles 1	Reference	Reference	Reference
Tertiles 2	2.09 (1.55, 2.84) < 0.0001	1. 41 (1.03, 1.94) 0.0325	1.33 (0.86, 2.07) 0.1985
Tertiles 3	2.84 (2.12, 3.80) < 0.0001	1.78 (1.31, 2.42) 0.0002	1.77 (1.13, 2.76) 0.0121
***P*** for trend	2.91 (2.16, 3.92) < 0.0001	1.85 (1.33, 2.56) 0.0002	1.90 (1.16, 3.12) 0.0112

Crude Model: No covariates were adjusted

Minimally adjusted model: Age, gender, and race were adjusted

Fully adjusted model: Age, gender, race, annual household income, education level, smoking status, drinking status, hypertension, diabetes, dietary supplements use, regular exercise, cancer, and cholesterol were adjusted

**Fig. 2:**
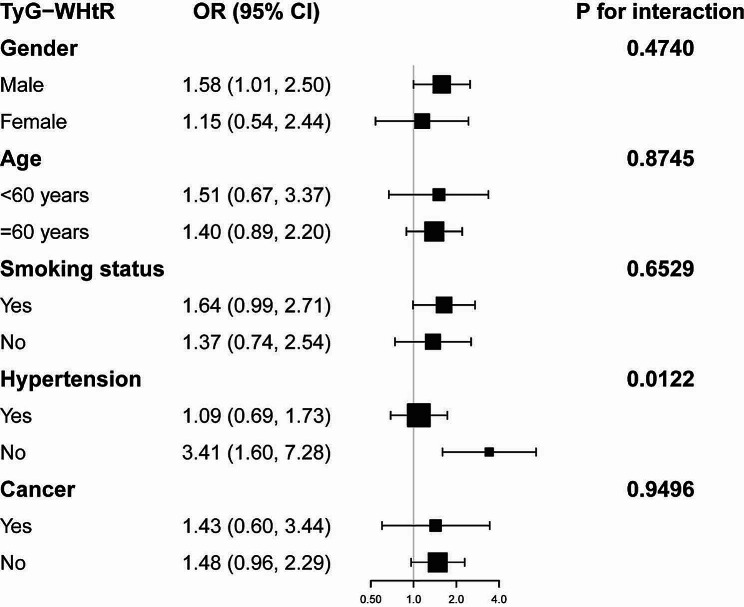
Subgroup analysis

Age, gender, race, education level, hypertension, dietary supplement use, and cholesterol level remained substantially linked to coronary heart disease risk in the fully adjusted models (Table 4). To determine whether these findings were applicable to the current population, we conducted a subgroup analysis by gender, age, smoking status, hypertension, and cancer. As shown in Fig. 2, there was a positive link between TyG-WHtR and coronary heart disease risk in both males (OR = 1.58; 95% CI: 1.01–2.50) and participants without hypertension (OR = 3.41, 95% CI: 1.60–7.28). The results of the interaction test demonstrated that there was no statistically significant difference in the association between TyG-WHtR and coronary heart disease according to gender, age, smoking status, or cancer, suggesting that these factors did not significantly affect the positive relation (*P* > 0.05 for interaction test).

**Table 4:** Multivariate analysis of associations between various variables and coronary heart disease

Variable	OR (95% CI)	*P* value
Age (year)	1.07 (1.05, 1.08)	< 0.0001
gender		
Female	Reference	
Male	1.81(1.23, 2.65)	0.0023
Race		
Mexican American	Reference	
Other Hispanic	2.81 (1.19, 6.62)	0.0181
Non-Hispanic White	3.22 (1.49, 6.94)	0.0029
Non-Hispanic Black	1.73 (0.74, 4.09)	0.2088
Other Races	1.30 (0.48, 3.56)	0.6058
Education level		
Less than 9th grade	Reference	
9-11th grade	0.39 (0.18,0.85)	0.0175
high school graduate AA degree College graduate or above	0.51 (0.26, 0.99) 0.56 (0.29, 1.07) 0.43(0.21, 0.85)	0.0466 0.0803 0.0158
Annual household income		
No	Reference	
Yes	0.93(0.59, 1.47)	0.7522
Smoking status		
No	Reference	
Yes	1.10 (0.78, 1.55)	0.6008
Drinking status		
No	Reference	
Yes	0.52 (0.07, 4.03)	0.5361
Hypertension		
No	Reference	
Yes	2.40 (1.63, 3.52)	< 0.0001
Diabetes		
No	Reference	
Yes	0.99 (0.64, 1.52)	0.9602
Dietary supplements use		
No	Reference	
Yes	1.47 (1.00, 2.15)	0.0498
Regular exercise		
No	Reference	
Yes	1.10 (0.78, 1.55)	0.5930
Cancer		
No	Reference	
Yes	0.95 (0.62, 1.45)	0.8164
Cholesterol (mmol/L)	0.59 (0.49, 0.71)	< 0.0001

Fully adjusted model: Age, gender, race, annual household income, education level, smoking status, drinking status, hypertension, diabetes, dietary supplements use, regular exercise, cancer, and cholesterol were adjusted

**Fig. 3:**
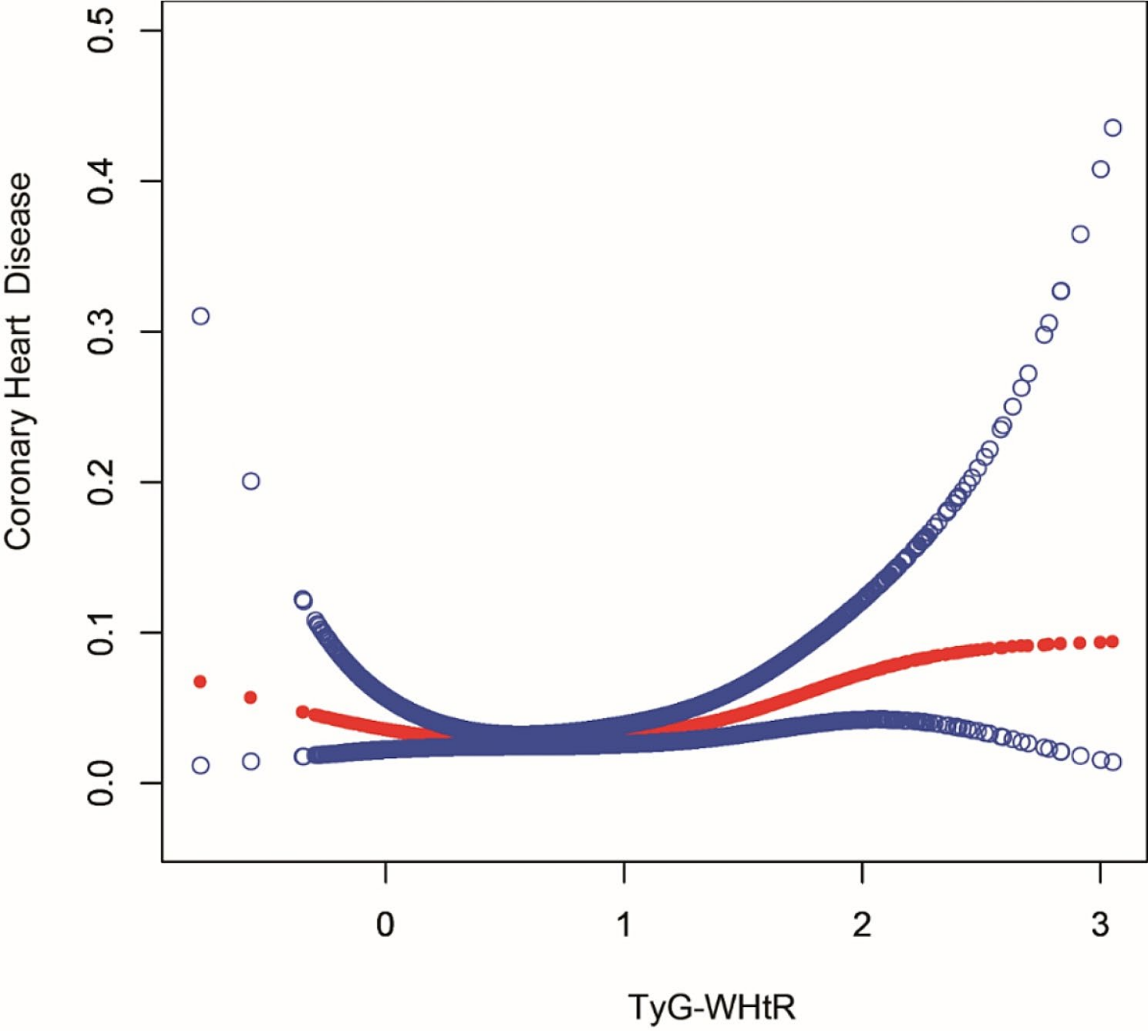
Smooth curve fitting. The red curve indicates the smooth curve fit between the variables; the two blue curves indicate the 95% confidence intervals of the fitted results

This nonlinear connection between TyG-WHtR and coronary heart disease risk was better characterized when all-variable smoothed curve fitting was considered. Smooth curve fitting revealed a U-shaped relation between TyG-WHtR and coronary heart disease (Fig. 3), with a log-likelihood ratio of 0.018 and a breakpoint of 0.36 (Table 5).

**Table 5:** Threshold effect

Outcome:	Coronary heart disease risk
Model I	
A straight-line effect	1.56 (1.05, 2.31)
Model II	
Fold points (K)	0.36
< K-segment effect 1	0.15 (0.03, 0.92)
> K-segment effect 2	1.93 (1.26, 2.94)
Effect size difference of 2 vs. 1	12.53 (1.79, 87.63)
Equation-predicted values at breakpoints	-3.83 (-4.08, -3.57)
Log likelihood ratio tests	0.018

Result variable: coronary heart disease

Exposure variables: TyG-WHtR

Results are expressed as OR (95%CI)

Adjusted for age, gender, race, annual household income, education level, smoking status, drinking status, hypertension, diabetes, dietary supplements use, regular exercise, cancer, and cholesterol

**Fig. 4:**
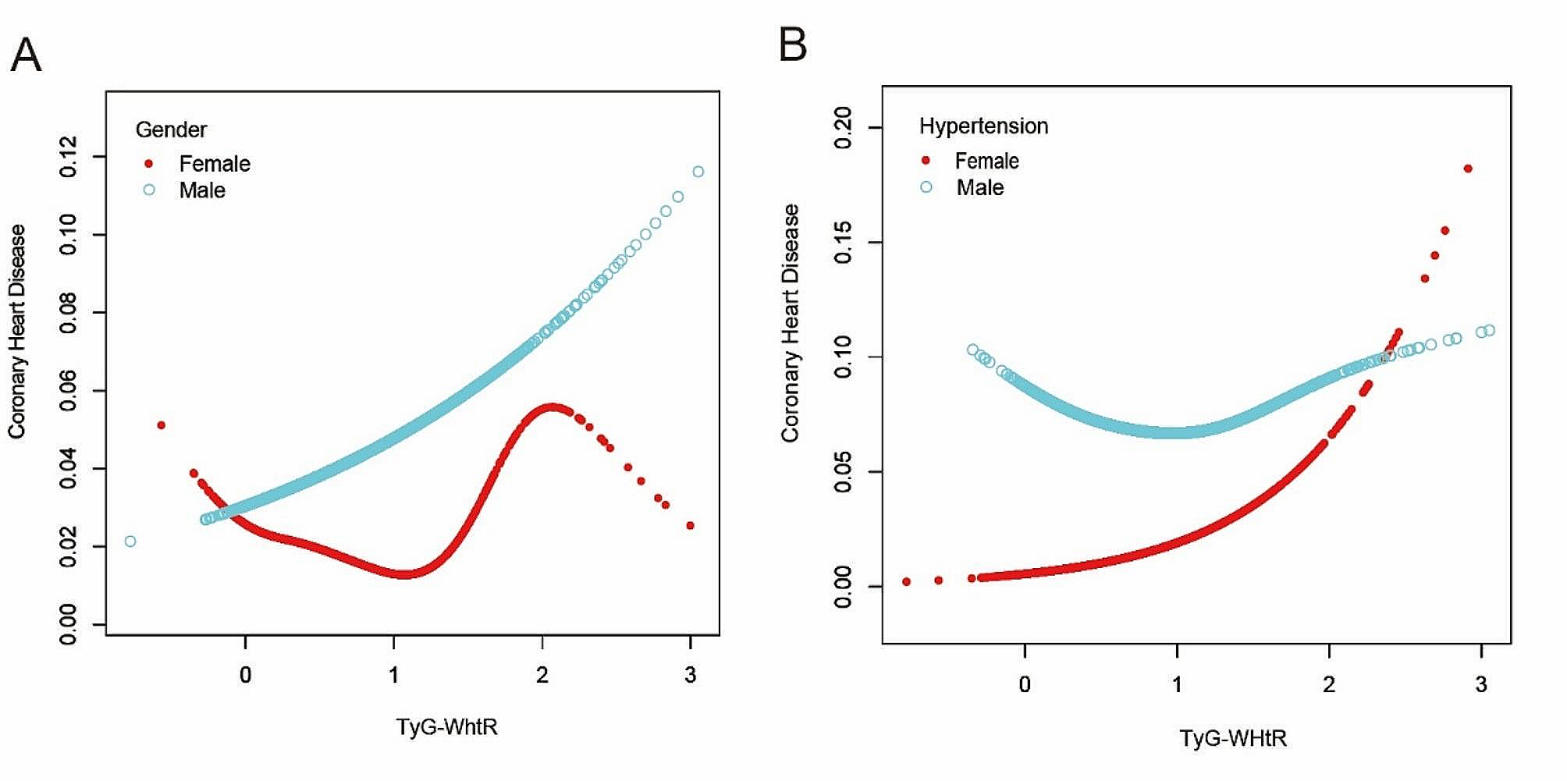
Smoothed curve fitting for subgroup analysis. **A:** Curve fitting with gender as the subgroup; **B:** Curve fitting with hypertension as the subgroup

Smoothed curves were subsequently constructed for the gender and hypertension subgroups based on the outcomes of the subgroup analyses. Surprisingly, TyG-WHtR was positively correlated with coronary heart disease risk in the subgroup of male but had a U-shaped association with coronary heart disease risk in the hypertension subgroup (Fig. 4).

## Discussion

To the best of our knowledge, this is the first study to evaluate the association between risk and TyG-WHtR in individuals with coronary heart disease. In this cross-sectional survey, higher continuous and categorical TyG-WHtR concentrations were associated with an increased risk of coronary heart disease. The proportion of individuals at high risk of coronary heart disease increased significantly with a gradual increase in TyG-WHtR. Other risk variables had no effect on this relation. The number of people who experienced this connection was consistent with the findings of the subgroup analyses and interaction tests. With an inflection point of 0.36, a U-shaped relation was observed between TyG-WHtR levels and coronary heart disease. Around the 0.36 inflection point, this investigation revealed a strong association between TyG-WHtR and coronary heart disease. In the highest TyG-WHtR tertile, the risk of coronary heart disease increased 1.77-fold (Tertiles 3 vs. Tertiles 1).

Insulin resistance is a significant risk factor for coronary heart disease, which can lead to vascular endothelial damage, the development of atherosclerotic plaques, and other conditions that accelerate the progression of coronary heart disease [23, 24]. Recently, the TyG index was employed to measure atherosclerosis in individuals with cardiovascular disease [25–27]. An observational research cohort study showed that TyG-WC was positively correlated with the risk of the first myocardial infarction. According to the multivariate-adjusted model, as TyG-WC quartiles increased, so did the risk of myocardial infarction [28]. This notion is supported by our data. Additionally, a cohort of observational studies showed that an elevated TyG index was linked to an increased likelihood of developing chronic kidney disease and that chronic kidney disease could be prevented by early metabolic factor intervention, which would lower the incidence of cardiovascular disease as well as prospective death [29]. According to one study, a higher TyG index was linked to an increased risk of adverse cardiovascular events in patients receiving percutaneous coronary intervention for ST-segment elevation myocardial infarction [14]. An increased TyG index is substantially linked to an increased risk of atherosclerosis and renal microvascular damage, according to a study by Zhao [30, 31]. WHtR is a straightforward and readily accessible marker of generalized and abdominal obesity. Relevant studies have demonstrated that WHtR is more effective in identifying cardiometabolic risk factors than is waist circumference and body mass index for abdominal fat deposition, which are significantly associated with coronary heart disease [32, 33]. Furthermore, WHtR is a better option than waist circumference for examining the association between obesity and cardiovascular disease in individuals with lower body mass indices [34]. Additionally, according to a meta-analysis of 52 cohort studies, shorter patients had a higher risk of developing coronary heart disease [19]. Thus, the current study investigated the risk correlations between TyG-WHtR and coronary heart disease. These results indicated a positive correlation between coronary heart disease and TyG-WHtR levels. A U-shaped relation with a breakpoint of 0.36 was also found between TyG-WHtR levels and coronary heart disease. The study results indicated a connection between elevated TyG-WHtR levels and an increased risk of coronary heart disease.

The precise mechanism by which TyG-WHtR is associated with cardiovascular disease remains unclear [1]. Atherosclerosis may develop owing to systemic lipid abnormalities caused by IR, such as elevated triglycerides, enhanced low-density lipemia, postprandial lipemia, and decreased high-density lipoprotein levels. This may be the fundamental mechanism of action [35]. In addition, reduced insulin activity in the established ischemic myocardium restricts glucose accessibility. This causes changes in the metabolism of fatty acids, which increase oxygen consumption in the heart and decrease its ability to compensate for non-infarcted regions [36]. Second, many adipokines and hormones produced by abdominal adipose tissue may cause endocrine-metabolic comorbidities [37]. Increased adipocyte size (hypertrophy) and number (hyperplasia) are associated with local and systemic chronic inflammation. Following the infiltration of inflammatory cells, inflammatory mediators and oxidative stressors are released, resulting in severe metabolic disorders that can directly affect the cardiovascular system and accelerate the development of atherosclerosis [38, 39]. The progression of coronary heart disease may be worsened by pathologic metabolic disturbances, such as hypertriglyceridemia, increased free fatty acids, adipose tissue emission of proinflammatory cytokines, hepatic IR and inflammation, amplified secretion of very-low-density lipoproteins, and impaired clearance of triglyceride-rich lipoproteins [37, 40].

### Study strengths and limitations

The findings of this study have significant implications for clinical practice. This study hypothesized that TyG-WHtR, an inexpensive and readily available marker, is associated with coronary heart disease risk. Therefore, prevention and treatment should be considered in patients with various risk factors. The data used in the analysis performed in this research were derived from the NHANES database, a well-designed, well-sampled program with the advantages of high representativeness, large datasets, and long-term monitoring. The anthropometric and laboratory data collected for this study were also of excellent quality. To conduct a relatively thorough analysis, this study used different methods: first, a large sample size and reliable data were used; second, covariate adjustments were made throughout the investigation of the relation between TyG-WHtR and coronary artery disease; and third, the exposure factors in this study were examined as categorical and continuous variables in two separate approaches. Smoothed curve fitting with a threshold effect analysis was employed to further investigate the nonlinear link between TyG-WHtR and coronary heart disease. The possibility of false positives was further decreased by the sensitivity analysis. As this was a cross-sectional study, it may have been influenced by other factors. However, thorough statistical corrections lessened the influence of confounding variables.

Nevertheless, this study has several limitations. First, this cross-sectional study examined the relation between TyG-WHtR and coronary heart disease. To further elucidate the connection between TyG-WHtR and coronary heart disease, several prospective studies, along with basic research, must be conducted at later time points. The data were collected at a specific point in time, and the lack of corresponding longitudinal data did not indicate a causal relation. Second, there is a possibility of bias because some indicators, such as recall, were partially collected through questionnaires rather than through objective measurement indications. Some variables, such as genetic and environmental factors, may still impact our results, even after several covariate adjustments. As a result, a significant number of further multidisciplinary investigations must be carried out to confirm our findings. Furthermore, cross-sectional studies cannot demonstrate temporal and causal correlations, and TyG-WHtR was evaluated only at baseline in this study. TyG-WHtR variations were not measured during the course of the inquiry. This study found a positive correlation between TyG-WHtR and coronary heart disease risk. However, further experiments are needed to confirm the mechanism underlying this correlation and the reduction of coronary heart disease risk by controlling the TyG-WHtR level in the clinic.

## Conclusions

In conclusion, this study demonstrated for the first time that elevated TyG-WHtR levels are associated with a higher risk of coronary heart disease in the United States. In this study, TyG-WHtR levels were associated with coronary heart disease risk in a “U-shaped” relation with a threshold value of 0.36. The results of this study provide a useful and convenient marker for early intervention of metabolic factors in people at a high risk of coronary heart disease.

### Electronic supplementary material

Below is the link to the electronic supplementary material.

Supplementary Material 1

Supplementary Material 2

## Data Availability

No datasets were generated or analysed during the current study.

## Abbreviations

TyG: Triglyceride glucose
TyG-WHtR: Triglyceride glucose-waist to height ratio
TyG-BMI: Triglyceride glucose-body mass index
TyG-WC: Triglyceride glucose-waist circumference
OR: Odds ratio
CI: Confidence interval
IR: Insulin resistance
NHANES: National Health and Nutrition Examination Survey
CHD: Coronary heart disease
