# Supplementary material for: Association between triglyceride glucose-waist to height ratio and coronary heart disease: a population-based study
Source: Lipids Health Dis. 2024 Jun 3;23:162. doi: 10.1186/s12944-024-02155-4 (PMC11145810; doi:10.1186/s12944-024-02155-4)
Supplement: Supplementary file 1 — Supplementary Material 1 [file 12944_2024_2155_MOESM1_ESM.pdf]

editage

Editing Certificate

This document certifies that the manuscript listed below has been edited to ensure language and grammar accuracy and is error free in these aspects. The edit was performed by professional editors at Editage, a brand of Cactus Communications. The author's core research ideas were not altered in any way during the editing process. The quality of the edit has been guaranteed, with the assumption that our suggested changes have been accepted and the text has not been further altered without the knowledge of our editors.

MANUSCRIPT TITLE

Association between triglyceride glucose-waist to height ratio and coronary heart disease: A population-based study

AUTHORS

Yangping Zhuang, Yu Wang, Peifen Sun, Jun Ke, Feng Chen

ISSUED ON

May 16, 2024

JOB CODE

HELIX_2780

THE EDITAGE Promise QUALITY GUARANTEE

Prabh Grewal
Senior Vice President - Editage

editage helping you get published

Since 2002, Editage has helped over 430,000 authors publish around 1.2 million research papers in scholarly journals across over 1000 disciplines through editorial, translation, transcription, and publication support services. Editage is a brand of Cactus Communications (cactusglobal.com), a science communication and technology company.

CACTUS.

GLOBAL :
+1(833) 979-0061 | request@editage.com

CHINA :
400-120-3020或021-6020-9400 |
fabiao@editage.cn

editage.com | editage.co.kr | editage.jp | editage.cn | editage.com.br | editage.com.tw | editage.de
