# Supplementary material for: Association between triglyceride glucose-waist to height ratio and coronary heart disease: a population-based study
Source: Lipids Health Dis. 2024 Jun 3;23:162. doi: 10.1186/s12944-024-02155-4 (PMC11145810; doi:10.1186/s12944-024-02155-4)
Supplement: Supplementary file 2 — Supplementary Material 2 [file 12944_2024_2155_MOESM2_ESM.pdf]

PAPER NAME

manuscript

AUTHOR

-

WORD COUNT

5905 Words

CHARACTER COUNT

34922 Characters

PAGE COUNT

28 Pages

FILE SIZE

674.6KB

SUBMISSION DATE

May 19, 2024 10:22 AM GMT+8

REPORT DATE

May 19, 2024 10:23 AM GMT+8

9% Overall Similarity

The combined total of all matches, including overlapping sources, for each database.

- 8% Internet database
- 8% Publications database
- Crossref database
- Crossref Posted Content database

Excluded from Similarity Report

- Bibliographic material
- Manually excluded text blocks

Association between triglyceride glucose-waist to height ratio and coronary heart disease: A population-based study

Yangping Zhuang[1,2†], Yu Wang[1,2†], Peifen Sun[1,2], Jun Ke[1,2*], Feng Chen[1,2*]

[1]Shengli Clinical Medical College of Fujian Medical University, Department of Emergence, Fujian Provincial Hospital, Fuzhou, China.

[2]Fujian Provincial Key Laboratory of Emergency Medicine, Fujian Emergency Medical Center, Fuzhou, China.

†These authors contributed equally to this work.

*Correspondence: 68223384@qq.com, cf9066@126.com.

Abstract

Background: The Triglyceride glucose (TyG) index-related indicators improve risk stratification by identifying individuals prone to atherosclerosis early in life. This study aimed to examine the relation between TyG-waist circumference-to-height ratio (TyG-WHtR) and coronary heart disease.

Methods: Data from four National Health and Nutrition Examination Surveys (NHANES) cycles between 2011 and 2018 were used for a cross-sectional study. The association between TyG-WHtR and coronary heart disease risk was examined using a multifactorial logistic regression model, and corresponding subgroup analyses were performed. Nonlinear correlations were analyzed using smooth curve fitting and threshold effects analysis. When nonlinear connections were discovered, appropriate inflection points were investigated using

recursive methods.

Results: TyG-WHtR and coronary heart disease were significantly positively correlated in the multifactorial logistic regression analysis. Subgroup analyses and interaction tests revealed that gender, age, smoking status, and cancer were not significantly associated with this correlation (***P*** for interaction>0.05). Furthermore, utilizing threshold effect analysis and smooth curve fitting, a nonlinear connection with an inflection point of 0.36 was observed between TyG-WHtR and coronary heart disease.

Conclusions: According to this study, the American population is far more likely to have coronary heart disease if they have higher TyG-WHtR levels.

Keywords

TyG-WHtR, coronary heart disease, NHANES, adults, cross-sectional analysis

Introduction

Cardiovascular disease, one of the conditions with the highest rates of morbidity and mortality globally, presents a public health challenge as well as a significant financial and psychological burden on patients and their families[1]. Approximately one-quarter of all deaths in the US are attributed to coronary heart disease annually[2]. Atherosclerosis and coronary heart disease are closely associated; in coronary heart disease, atherosclerotic plaques form in the coronary arteries that supply the myocardium, reducing blood flow perfusion to the heart[3]. Metabolic syndrome plays a significant role in the development of coronary heart disease, with varying levels of correlation between the incidence and prevalence of coronary heart disease, suggesting

that there may be specific common disease progressions between them. Diabetes mellitus, dyslipidemia, and metabolic dysfunction, a collection of concurrent conditions characterized by insulin resistance[4, 5], are of great interest in actively investigating the precise mechanisms by which metabolic dysfunction influences the onset of coronary heart disease. Determining relevant pathways is crucial for diagnosing, treating, and preventing coronary heart disease.

Insulin resistance (IR) is a condition in which the body is less sensitive and responsive to the effects of insulin. Patients with IR are more likely to develop various metabolic diseases, including dyslipidemia, hypertension, and abnormal blood sugar levels. These conditions increase the risk of atherosclerosis, inflammation, and coagulation abnormalities and are also significantly associated with a worse prognosis for cardiovascular disease[1, 6-8]. The TyG index is a correlative indicator of metabolic dysfunction that determines the levels of glucose and triglycerides in fasting blood. The TyG index has recently gained recognition as a reliable and user-friendly alternative to IR with significant therapeutic implications for identifying metabolic dysfunction[9]. The TyG index outperformed the homeostasis model assessment of insulin resistance in predicting coronary artery calcification[10]. It is substantially correlated with carotid atherosclerosis, even after controlling for conventional cardiovascular risk factors[11]. In individuals with type 2 diabetes, Chen et al. recently noted an independent correlation between the TyG index and severe coronary artery stenosis[12]. The TyG index is linked to an increased risk of cardiovascular disease, atherosclerosis, and coronary artery calcification[10, 13]. The TyG-related index is a more reliable predictor of IR and cardiometabolic risk than is the TyG index alone[14, 15]. Additionally, correlative indices, such

as Triglyceride glucose-body mass index (TyG-BMI), Triglyceride glucose-waist circumference (TyG-WC), and Triglyceride glucose-waist circumference-to-height ratio (TyG-WHtR), which incorporate anthropometric measurements of obesity in addition to TyG, are more accurate predictors of Insulin resistance and cardiometabolic risk than is the TyG index alone[16-19]. Furthermore, the integrated association score of TyG and anatomical indicators of obesity is a more precise indicator of the degree of insulin resistance. Therefore, early monitoring of insulin resistance in obese individuals is crucial to avoid a poor prognosis in coronary heart disease.

To the best of our knowledge, there is inadequate data on the association between TyG-WHtR levels and coronary heart disease. Identifying practical and helpful markers that allow early intervention in metabolic factors in individuals at high risk of coronary heart disease is crucial. Thus, our investigation aimed to examine the association between TyG-WHtR indices and the risk of coronary heart disease using a large-scale, cross-sectional, population-based study.

Methods

Data sources and study population

The Centers for Disease Control and Prevention conducted the NHANES study of the ambulatory population in the United States to evaluate the health, nutritional status, and lifestyle of Americans. The NHANES employs a sophisticated multistage stratified sampling strategy and releases data biennially. The National Center for Health Statistics Research Ethics Review Board approved all technique utilized in the NHANES study. All survey respondents

provided written informed consent. During the survey, participants completed several questions and pertinent tests. The five main components of the NHANES database are screening, diet, laboratory, questionnaire, and demographic data. This analysis used data from four NHANES cycles from 2011 to 2018.

Studies on research designs have been published[20]. Overall, 39,156 participants were identified from the NHANES statistics spanning 2011 to 2018. The exclusion criteria included: 16,539 participants under 20 years of age, 13,429 participants with missing TyG and WHtR data, and 37 participants with missing coronary heart disease data. Ultimately, we analyzed the outcome data of 9,151 participants (Figure 1).

Figure 1: Flow chart.

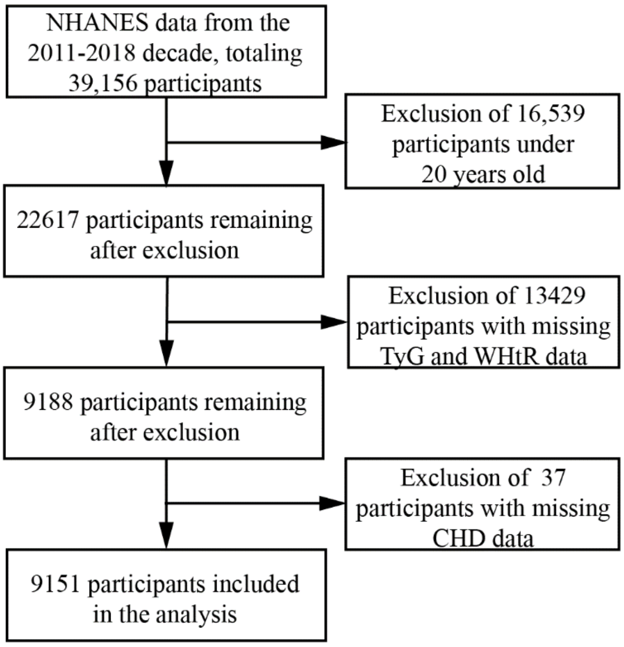

Definitions of TyG-WHtR and coronary heart disease

The TyG index measures triglyceride and fasting blood glucose levels to quantify insulin resistance. The following formula was used to calculate the correlation index: TyG=Ln[fasting blood glucose(mg/dL)×fasting triglyceride(mg/dL)/2][21]; WHtR=waist circumference/height; TyG-WHtR=TyG×WHtR[18]. The exposure variable in our study was TyG-WHtR.

A trained interviewer asked patients a series of questions about their coronary heart disease history. Participants were asked, “Has a doctor or other health professional ever told you that you had coronary heart disease?” They responded with either “yes” or “no.” Those who were unsure or declined to respond were considered absent. Coronary heart disease was the outcome variable in our analysis[22].

Study variables

The covariates included age, gender, race, annual household income, education level, smoking status, drinking status, hypertension, diabetes, dietary supplement use, regular exercise, cancer, and cholesterol levels. Race was categorized as Mexican American, Other Hispanic, Non-Hispanic White, Non-Hispanic Black, or Other. Educational level was designated as less than 9th grade, 9th–11th grade, high school graduate, AA degree, or college graduate or above. Annual household income was grouped with a threshold of USD 100,000. Smoking status was determined by having smoked more than 100 cigarettes in a lifetime. Drinking status was defined based on consuming more than 12 drinks per year, with those answering “yes” considered drinkers. Medical professionals confirmed diagnoses of hypertension, diabetes, and

cancer. Waist circumference and height, vital measurements taken during a medical checkup, along with fasting blood glucose, triglyceride, and total cholesterol levels, were assessed using relevant laboratory tests. For more detailed information on how these variables were measured, please refer to visit the NHANES website: www.cdc.gov/nchs/nhanes/.

Statistical analysis

All statistical analyses were performed by considering intricate multistage cluster surveys and using proper NHANES sampling weights in compliance with the Centers for Disease Control and Prevention criteria. The participants were divided into groups based on the presence of TyG-WHtR and coronary heart disease. Continuous data are represented as mean ± standard deviation, and categorical variables are expressed as frequency percentages[20]. All variables were evaluated for variance using the chi-square test, nonparametric Kruskal–Wallis test, or analysis of variance. The participants were divided into groups depending on the presence of TyG-WHtR and coronary heart disease. To identify any possible correlation between the TyG-WHtR index (continuous or tertile) and coronary heart disease, multivariate logistic regression analysis was used to determine the odds ratio (OR) and 95% confidence interval (CI) in three distinct models. Using multivariate regression analysis, the independent relation between TyG-WHtR and coronary heart disease was examined using three distinct models. The crude model was not adjusted for correlation, the minimally adjusted model was adjusted for age, gender, and race, and the fully adjusted model was adjusted for education level, smoking status, drinking status, hypertension, diabetes, dietary supplement use, regular exercise, cancer, and cholesterol. The model was fully adjusted for age, gender, and race. The subgroup analyses of

TyG-WHtR and coronary artery disease were stratified using criteria based on gender, age, smoking status, hypertension, and cancer. A threshold effect analysis was used to examine the relation and inflection points between TyG-WHtR and coronary heart disease. Finally, we characterized the nonlinear connection between TyG-WHtR and coronary heart disease risk using smooth curve fitting. R Studio (version 4.3.1) and EmpowerStats (version 2.0) were used for statistical analysis. Statistical significance was set at $P<0.05$.

Results

Baseline Characteristics of Participants

A total of 9,151 individuals were included in the study, with a mean age of 49.57±17.38 years. Among the participants, 51.31% were male and 48.69% were female. The racial composition was as follows: 13.51% Mexican Americans, 10.83% Other Hispanics, 37.92% Non-Hispanic White, 21.21% Non-Hispanic Black, and 16.53% from other racial backgrounds. Coronary heart disease was diagnosed in 4.04% of the participants.

The clinical features of the individuals are listed in Table 1, arranged according to the TyG-WHtR tertiles. The tertiles were statistically significant for age, gender, race, annual household income, educational level, smoking status, drinking status, hypertension, diabetes, cancer, cholesterol, and coronary heart disease ($P<0.05$). Participants in the Tertiles 3 group tended to be older, male, and Non-Hispanic White, with an annual household income under $100,000, a higher education level, and higher cholesterol levels.

Table 1: Characteristics of the study population based on TyG-WHtR.

**TyG-WHtR**	**Tertiles 1 (N=3050)**	**Tertiles 2 (N=3050)**	**Tertiles 3 (N=3051)**	***P* value**
Age (years)	43.91 ± 17.65	51.01 ± 17.21	53.79 ± 15.71	<0.001
gender (%)				<0.001
Male	42.46%	51.11%	52.51%	
Female	57.54%	48.89%	47.49%	
Race (%)				<0.001
Mexican American	8.82%	13.84%	17.86%	
Other Hispanic	7.90%	11.74%	12.85%	
Non-Hispanic White	35.05%	37.21%	41.49%	
Non-Hispanic Black	29.70%	20.52%	13.41%	
Other Races	18.52%	16.69%	14.39%	
Annual household income (%)				<0.001
Yes	22.10%	19.16%	13.40%	
No	77.90%	80.84%	86.60%	
Education level (%)				<0.001
Less than 9th grade	5.35%	9.05%	12.46%	
9-11th grade	11.02%	13.34%	15.25%	
high school graduate	21.03%	22.26%	22.20%	
AA degree	31.30%	30.23%	29.68%	
College graduate or above	31.30%	25.11%	20.40%	
Smoking status (%)				<0.001
Yes	37.16%	44.69%	48.28%	
No	62.84%	55.31%	51.72%	
Drinking status (%)				0.013
Yes	1.48%	1.63%	2.68%	
No	98.52%	98.37%	97.32%	

Hypertension (%)				<0.001
Yes	24.35%	35.70%	50.56%	
No	75.65%	64.30%	49.44%	
Diabetes (%)				<0.001
Yes	3.68%	10.44%	28.60%	
No	96.32%	89.56%	71.40%	
Dietary supplements use (%)				0.368
Yes	51.87%	53.05%	53.64%	
No	48.13%	46.95%	46.36%	
Regular exercise (%)				0.069
Yes	21.59%	19.84%	19.31%	
No	78.41%	80.16%	80.69%	
Cancer (%)				<0.001
Yes	6.82%	9.54%	10.96%	
No	93.18%	90.46%	89.04%	
Cholesterol (mmol/L)	4.55 ± 0.91	4.95 ± 1.03	5.21 ± 1.17	<0.001
Coronary heart disease (%)				<0.001
Yes	2.10%	4.30%	5.74%	
No	97.90%	95.70%	94.26%	

Mean ± SD for continuous variables: the ***P*** value was calculated by the weighted linear regression model.

(%) For categorical variables, the ***P*** value was calculated using the chi-square test.

The clinical features of the individuals are listed in Table 2, according to whether they had coronary heart disease. Age, gender, race, annual household income, educational level, smoking status, hypertension, diabetes, dietary supplement use, regular exercise, cancer, cholesterol level, and the TyG-WHtR were significantly associated with the presence or absence of coronary heart disease (***P***<0.05). Patients with coronary heart disease tended to be older, male,

Non-Hispanic White, with an annual household income under $100,000, educated to an AA degree, with smoking status, hypertension, dietary supplement use, and higher TyG-WHtR levels than those without the condition.

Table 2: Characteristics of the study population based on coronary heart disease.

	**Coronary heart disease (N=370)**	**Non-coronary heart disease (N=8781)**	***P* value**
Age (years)	68.55 ± 10.54	48.77 ± 17.15	<0.001
Gender (%)			<0.001
Male	64.86%	48.01%	
Female	35.14%	51.99%	
Race (%)			<0.001
Mexican American	7.57%	13.76%	
Other Hispanic	9.73%	10.88%	
Non-Hispanic White	58.65%	37.05%	
Non-Hispanic Black	14.05%	21.51%	
Other Races	10.00%	16.81%	
Annual household income (%)			0.002
Yes	12.03%	18.48%	
No	87.97%	81.52%	
Education level (%)			0.001
Less than 9th grade	14.59%	8.72%	
9-11th grade	13.51%	13.19%	
high school graduate	21.35%	21.85%	
AA degree	30.00%	30.42%	
College graduate or above	20.54%	25.82%	
Smoking status (%)			<0.001

Yes	59.19%	42.71%	
No	40.81%	57.29%	
Drinking status (%)			0.156
Yes	0.52%	1.95%	
No	99.48%	98.05%	
Hypertension (%)			<0.001
Yes	77.51%	35.16%	
No	22.49%	64.84%	
Diabetes (%)			<0.001
Yes	39.15%	13.14%	
No	60.85%	86.86%	
Dietary supplements use (%)			<0.001
Yes	68.92%	52.18%	
No	31.08%	47.82%	
Regular exercise (%)			0.036
Yes	15.95%	20.43%	
No	84.05%	79.57%	
Cancer (%)			<0.001
Yes	22.16%	8.56%	
No	77.84%	91.44%	
Cholesterol (mmol/L)	4.41 ± 1.17	4.92 ± 1.06	<0.001
TyG-WHtR	0.94 ± 0.53	0.73 ± 0.47	<0.001

Mean±SD for continuous variables: the ***P*** value was calculated by the weighted linear regression model.

(%) For categorical variables, the ***P*** value was calculated using the chi-square test.

Relation between TyG-WHtR and coronary heart disease

The findings of the multivariate regression analysis of TyG-WHtR and coronary heart disease are shown in Table 3. The fully adjusted model revealed that the association between TyG-

WHtR and coronary heart disease was significantly positive (OR=1.83; 95% CI: 1.28-2.62, ***P***=0.0008), indicating a higher TyG-WHtR value increases the odds of developing coronary heart disease. This association was evident in both the crude (OR=2.26; 95% CI: 1.87-2.72, ***P***<0.0001) and minimally adjusted model (OR=1.85; 95% CI: 1.48-2.32, ***P***<0.0001). For additional sensitivity analyses, TyG-WHtR was converted from continuous to categorical variables (Tertiles 1, 2, and 3). In the fully adjusted model, participants in Tertile 3 had a 77% higher risk of coronary heart disease than those in Tertile 1 (OR=1.77; 95% CI: 1.13-2.76, ***P***=0.0121). Furthermore, all three models had trend tests with ***P*** values of < 0.05, indicating statistical significance.

Table 3: Association between TyG-WHtR and coronary heart disease.

	**OR (95%CI), *P*-value**		
	**Crude model**	**Minimally adjusted model**	**Fully adjusted model**
TyG-WHtR	2.26 (1.87, 2.72)	1.85 (1.48, 2.32)	1.83 (1.28, 2.62)
	<0.0001	<0.0001	0.0008
TyG-WHtR (Tertiles)			
Tertiles 1	Reference	Reference	Reference
Tertiles 2	2.09 (1.55, 2.84)	1. 41 (1.03, 1.94)	1.33 (0.86, 2.07)
	<0.0001	0.0325	0.1985
Tertiles 3	2.84 (2.12, 3.80)	1.78 (1.31, 2.42)	1.77 (1.13, 2.76)
	<0.0001	0.0002	0.0121
P for trend	2.91 (2.16, 3.92)	1.85 (1.33, 2.56)	1.90 (1.16, 3.12)
	<0.0001	0.0002	0.0112

Crude Model: No covariates were adjusted.

Minimally adjusted model: Age, gender, and race were adjusted.

Fully adjusted model: Age, gender, race, annual household income, education level, smoking status, drinking status, hypertension, diabetes, dietary supplements use, regular exercise, cancer, and cholesterol were adjusted.

Age, gender, race, education level, hypertension, dietary supplement use, and cholesterol level remained substantially linked to coronary heart disease risk in the fully adjusted models (Table 4). To determine whether these findings were applicable to the current population, we conducted a subgroup analysis by gender, age, smoking status, hypertension, and cancer. As shown in Figure 2, there was a positive link between TyG-WHtR and coronary heart disease risk in both males (OR=1.58; 95% CI: 1.01-2.50) and participants without hypertension (OR=3.41, 95% CI: 1.60-7.28). The results of the interaction test demonstrated that there was no statistically significant difference in the association between TyG-WHtR and coronary heart disease according to gender, age, smoking status, or cancer, suggesting that these factors did not significantly affect the positive relation (***P***>0.05 for interaction test).

Table 4: Multivariate analysis of associations between various variables and coronary heart disease.

Variable	OR (95% CI)	*P* value
Age (year)	1.07 (1.05, 1.08)	<0.0001
gender		
Female	Reference	
Male	1.81(1.23, 2.65)	0.0023
Race		
Mexican American	Reference	
Other Hispanic	2.81 (1.19, 6.62)	0.0181
Non-Hispanic White	3.22 (1.49, 6.94)	0.0029
Non-Hispanic Black	1.73 (0.74, 4.09)	0.2088
Other Races	1.30 (0.48, 3.56)	0.6058
Education level		
Less than 9th grade	Reference	
9-11th grade	0.39 (0.18,0.85)	0.0175

high school graduate	0.51 (0.26, 0.99)	0.0466
AA degree	0.56 (0.29, 1.07)	0.0803
College graduate or above	0.43(0.21, 0.85)	0.0158
Annual household income		
No	Reference	
Yes	0.93(0.59, 1.47)	0.7522
Smoking status		
No	Reference	
Yes	1.10 (0.78, 1.55)	0.6008
Drinking status		
No	Reference	
Yes	0.52 (0.07, 4.03)	0.5361
Hypertension		
No	Reference	
Yes	2.40 (1.63, 3.52)	<0.0001
Diabetes		
No	Reference	
Yes	0.99 (0.64, 1.52)	0.9602
Dietary supplements use		
No	Reference	
Yes	1.47 (1.00, 2.15)	0.0498
Regular exercise		
No	Reference	
Yes	1.10 (0.78, 1.55)	0.5930
Cancer		
No	Reference	
Yes	0.95 (0.62, 1.45)	0.8164
Cholesterol (mmol/L)	0.59 (0.49, 0.71)	<0.0001

Fully adjusted model: Age, gender, race, annual household income, education level, smoking status, drinking status, hypertension, diabetes, dietary supplements use, regular exercise, cancer, and cholesterol were adjusted.

Figure 2: Subgroup analysis.

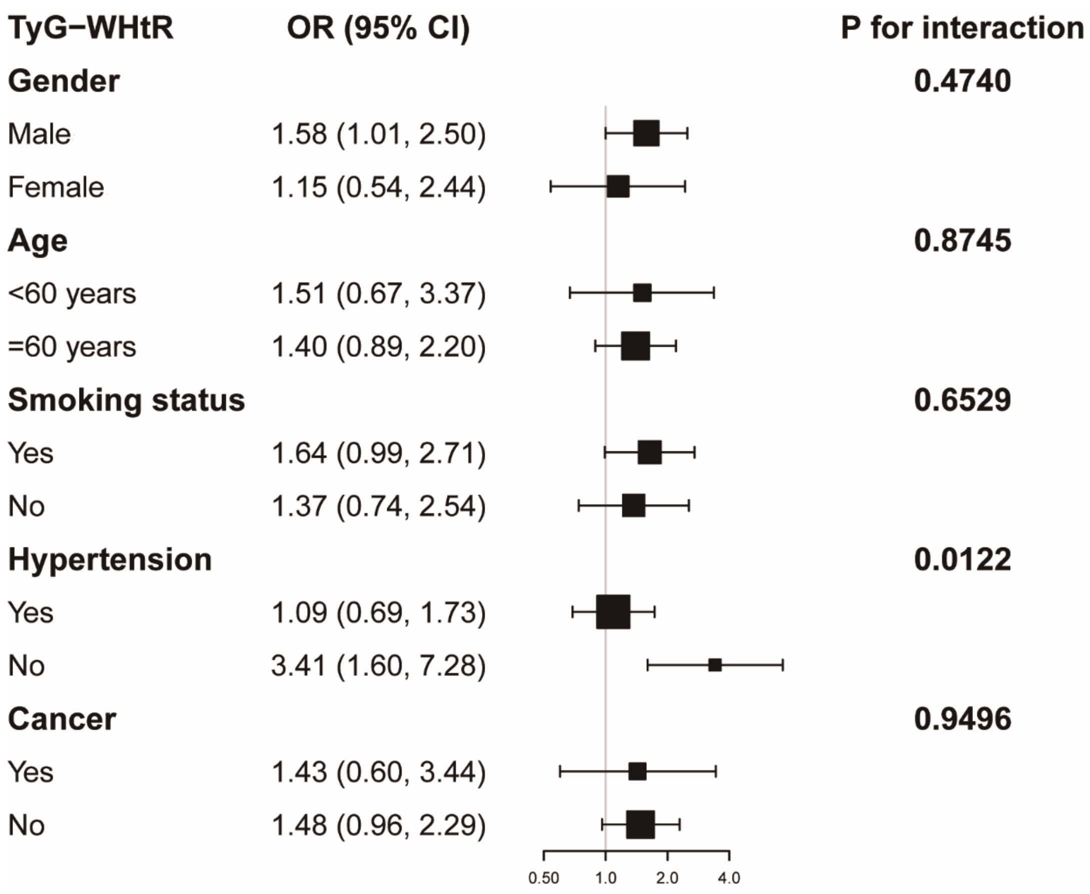

This nonlinear connection between TyG-WHtR and coronary heart disease risk was better characterized when all-variable smoothed curve fitting was considered. Smooth curve fitting revealed a U-shaped relation between TyG-WHtR and coronary heart disease (Figure 3), with a log-likelihood ratio of 0.018 and a breakpoint of 0.36 (Table 5).

Figure 3: Smooth curve fitting. The red curve indicates the smooth curve fit between the variables; the two blue curves indicate the 95% confidence intervals of the fitted results.

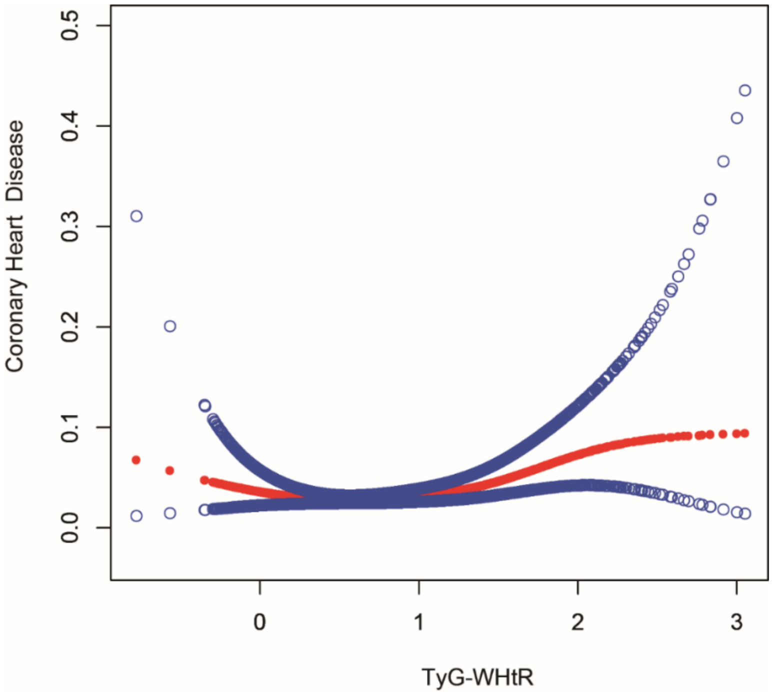

Table 5: Threshold effect.

Outcome:	Coronary heart disease risk
Model I	
A straight-line effect	1.56 (1.05, 2.31)
Model II	
Fold points (K)	0.36
< K-segment effect 1	0.15 (0.03, 0.92)
> K-segment effect 2	1.93 (1.26, 2.94)
Effect size difference of 2 vs. 1	12.53 (1.79, 87.63)
Equation-predicted values at breakpoints	-3.83 (-4.08, -3.57)
Log likelihood ratio tests	0.018

Result variable: coronary heart disease.
Exposure variables: TyG-WHtR.
Results are expressed as OR (95%CI).
Adjusted for age, gender, race, annual household income, education level, smoking status, drinking status, hypertension, diabetes, dietary supplements use, regular exercise, cancer, and cholesterol.

Smoothed curves were subsequently constructed for the gender and hypertension subgroups based on the outcomes of the subgroup analyses. Surprisingly, TyG-WHtR was positively correlated with coronary heart disease risk in the subgroup of male but had a U-shaped

association with coronary heart disease risk in the hypertension subgroup (Figure 4).

Figure 4: Smoothed curve fitting for subgroup analysis. A: Curve fitting with gender as the subgroup; B: Curve fitting with hypertension as the subgroup.

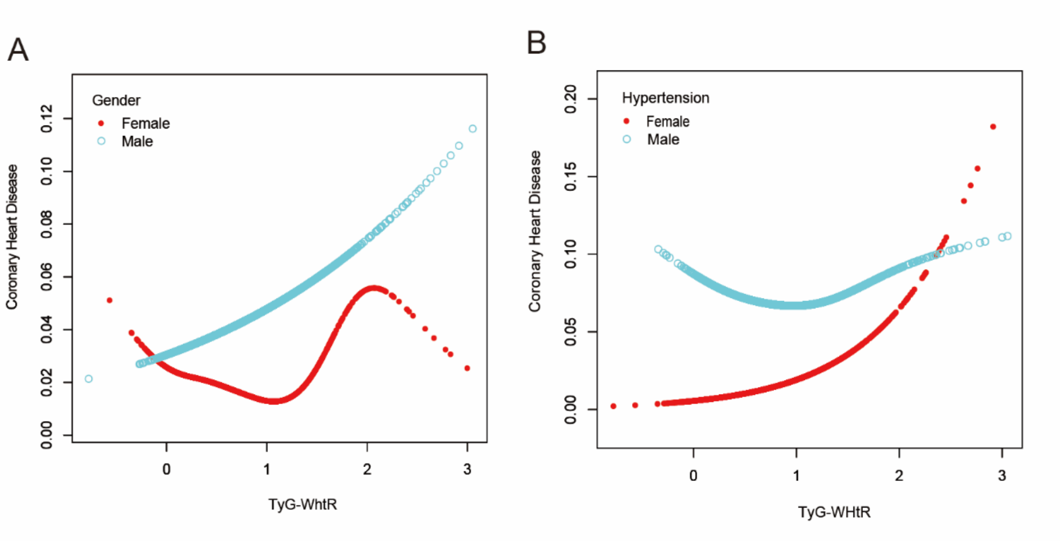

Discussion

To the best of our knowledge, this is the first study to evaluate the association between risk and TyG-WHtR in individuals with coronary heart disease. In this cross-sectional survey, higher continuous and categorical TyG-WHtR concentrations were associated with an increased risk of coronary heart disease. The proportion of individuals at high risk of coronary heart disease increased significantly with a gradual increase in TyG-WHtR. Other risk variables had no effect on this relation. The number of people who experienced this connection was consistent with the findings of the subgroup analyses and interaction tests. With an inflection point of 0.36, a U-shaped relation was observed between TyG-WHtR levels and coronary heart disease. Around the 0.36 inflection point, this investigation revealed a strong association between TyG-WHtR and coronary heart disease. In the highest TyG-WHtR tertile, the risk of coronary heart disease increased 1.77-fold (Tertiles 3 vs Tertiles 1).

Insulin resistance is a significant risk factor for coronary heart disease, which can lead to vascular endothelial damage, the development of atherosclerotic plaques, and other conditions that accelerate the progression of coronary heart disease [23, 24]. Recently, the TyG index was employed to measure atherosclerosis in individuals with cardiovascular disease[25-27]. An observational research cohort study showed that TyG-WC was positively correlated with the risk of the first myocardial infarction. According to the multivariate-adjusted model, as TyG-WC quartiles increased, so did the risk of myocardial infarction[28]. This notion is supported by our data. Additionally, a cohort of observational studies showed that an elevated TyG index was linked to an increased likelihood of developing chronic kidney disease and that chronic kidney disease could be prevented by early metabolic factor intervention, which would lower the incidence of cardiovascular disease as well as prospective death[29]. According to one study, a higher TyG index was linked to an increased risk of adverse cardiovascular events in patients receiving percutaneous coronary intervention for ST-segment elevation myocardial infarction[14]. An increased TyG index is substantially linked to an increased risk of atherosclerosis and renal microvascular damage, according to a study by Zhao[30, 31]. WHtR is a straightforward and readily accessible marker of generalized and abdominal obesity. Relevant studies have demonstrated that WHtR is more effective in identifying cardiometabolic risk factors than is waist circumference and body mass index for abdominal fat deposition, which are significantly associated with coronary heart disease [32, 33]. Furthermore, WHtR is a better option than waist circumference for examining the association between obesity and cardiovascular disease in individuals with lower body mass indices[34]. Additionally,

according to a meta-analysis of 52 cohort studies, shorter patients had a higher risk of developing coronary heart disease [19]. Thus, the current study investigated the risk correlations between TyG-WHtR and coronary heart disease. These results indicated a positive correlation between coronary heart disease and TyG-WHtR levels. A U-shaped relation with a breakpoint of 0.36 was also found between TyG-WHtR levels and coronary heart disease. The study results indicated a connection between elevated TyG-WHtR levels and an increased risk of coronary heart disease.

The precise mechanism by which TyG-WHtR is associated with cardiovascular disease remains unclear [1]. Atherosclerosis may develop owing to systemic lipid abnormalities caused by IR, such as elevated triglycerides, enhanced low-density lipemia, postprandial lipemia, and decreased high-density lipoprotein levels. This may be the fundamental mechanism of action[35]. In addition, reduced insulin activity in the established ischemic myocardium restricts glucose accessibility. This causes changes in the metabolism of fatty acids, which increase oxygen consumption in the heart and decrease its ability to compensate for non-infarcted regions[36]. Second, many adipokines and hormones produced by abdominal adipose tissue may cause endocrine-metabolic comorbidities[37]. Increased adipocyte size (hypertrophy) and number (hyperplasia) are associated with local and systemic chronic inflammation. Following the infiltration of inflammatory cells, inflammatory mediators and oxidative stressors are released, resulting in severe metabolic disorders that can directly affect the cardiovascular system and accelerate the development of atherosclerosis[38, 39]. The progression of coronary heart disease may be worsened by pathologic metabolic disturbances,

such as hypertriglyceridemia, increased free fatty acids, adipose tissue emission of proinflammatory cytokines, hepatic IR and inflammation, amplified secretion of very-low-density lipoproteins, and impaired clearance of triglyceride-rich lipoproteins[37, 40].

Study strengths and limitations

The findings of this study have significant implications for clinical practice. This study hypothesized that TyG-WHtR, an inexpensive and readily available marker, is associated with coronary heart disease risk. Therefore, prevention and treatment should be considered in patients with various risk factors. The data used in the analysis performed in this research were derived from the NHANES database, a well-designed, well-sampled program with the advantages of high representativeness, large datasets, and long-term monitoring. The anthropometric and laboratory data collected for this study were also of excellent quality. To conduct a relatively thorough analysis, this study used different methods: first, a large sample size and reliable data were used; second, covariate adjustments were made throughout the investigation of the relation between TyG-WHtR and coronary artery disease; and third, the exposure factors in this study were examined as categorical and continuous variables in two separate approaches. Smoothed curve fitting with a threshold effect analysis was employed to further investigate the nonlinear link between TyG-WHtR and coronary heart disease. The possibility of false positives was further decreased by the sensitivity analysis. As this was a cross-sectional study, it may have been influenced by other factors. However, thorough statistical corrections lessened the influence of confounding variables.

Nevertheless, this study has several limitations. First, this cross-sectional study examined the relation between TyG-WHtR and coronary heart disease. To further elucidate the connection between TyG-WHtR and coronary heart disease, several prospective studies, along with basic research, must be conducted at later time points. The data were collected at a specific point in time, and the lack of corresponding longitudinal data did not indicate a causal relation. Second, there is a possibility of bias because some indicators, such as recall, were partially collected through questionnaires rather than through objective measurement indications. Some variables, such as genetic and environmental factors, may still impact our results, even after several covariate adjustments. As a result, a significant number of further multidisciplinary investigations must be carried out to confirm our findings. Furthermore, cross-sectional studies cannot demonstrate temporal and causal correlations, and TyG-WHtR was evaluated only at baseline in this study. TyG-WHtR variations were not measured during the course of the inquiry. This study found a positive correlation between TyG-WHtR and coronary heart disease risk. However, further experiments are needed to confirm the mechanism underlying this correlation and the reduction of coronary heart disease risk by controlling the TyG-WHtR level in the clinic.

Conclusions

In conclusion, this study demonstrated for the first time that elevated TyG-WHtR levels are associated with a higher risk of coronary heart disease in the United States. In this study, TyG-WHtR levels were associated with coronary heart disease risk in a "U-shaped" relation with a threshold value of 0.36. The results of this study provide a useful and convenient marker for early intervention of metabolic factors in people at a high risk of coronary heart disease.

List of abbreviations

TyG: Triglyceride glucose; TyG-WHtR: Triglyceride glucose-waist to height ratio; TyG-BMI: Triglyceride glucose-body mass index; TyG-WC: Triglyceride glucose-waist circumference; OR: Odds ratio; CI: Confidence interval; IR: Insulin resistance; NHANES: National Health and Nutrition Examination Survey; CHD: Coronary heart disease.

Ethics approval and consent to participate

The National Center for Health Statistics Institutional Ethics Review Board examined and authorized studies involving human subjects, and all participants gave their written informed consent to participate in the study after agreeing to the survey and giving their permission in writing.

Consent for publication

Not applicable.

Availability of data and materials

In this study, publicly accessible datasets were evaluated. You may get this data here: www.cdc.gov/nchs/nhanes/(accessed on 15 February 2024).

Competing interests

The authors declare that the research was conducted without any commercial or financial

relationships that could be construed as a potential conflict of interest.

Funding

This work was Sponsored by Fujian provincial health technology project (NO. 2022ZD01008).

Authors' contributions

Conceptualization, ZY and WY; Formal analysis, ZY and WY; Methodology, ZY, WY, SP and KJ; Software, WY, SP and KJ; Supervision, KJ and CF; Visualization, KJ and CF; Writing-original draft, ZY and WY; Writing-review & editing, ZY, WY, SP, KJ and CF. All authors read and approved the final manuscript

Acknowledgements

We thank the National Center for Health Statistics of the Centers for Disease Control for Health Statistics employees for organizing, compiling, and developing the NHANES data and building the public database.

References

1. Tao LC, Xu JN, Wang TT, Hua F, Li JJ: **Triglyceride-glucose index as a marker in cardiovascular diseases: landscape and limitations.** *Cardiovasc Diabetol* 2022, **21**:68.
2. Tsao CW, Aday AW, Almarzooq ZI, Alonso A, Beaton AZ, Bittencourt MS, Boehme AK, Buxton AE, Carson AP, Commodore-Mensah Y, et al: **Heart Disease and Stroke Statistics-2022 Update: A Report From the American Heart Association.** *Circulation* 2022, **145**:e153-e639.
3. Liang X, Huang Y, Han X: **Associations between coronary heart disease and risk of cognitive impairment: A meta-analysis.** *Brain Behav* 2021,

11:e02108.

4. Powell-Wiley TM, Poirier P, Burke LE, Despres JP, Gordon-Larsen P, Lavie CJ, Lear SA, Ndumele CE, Neeland IJ, Sanders P, et al: **Obesity and Cardiovascular Disease: A Scientific Statement From the American Heart Association.** *Circulation* 2021, **143**:e984-e1010.
5. Dugani SB, Moorthy MV, Li C, Demler OV, Alsheikh-Ali AA, Ridker PM, Glynn RJ, Mora S: **Association of Lipid, Inflammatory, and Metabolic Biomarkers With Age at Onset for Incident Coronary Heart Disease in Women.** *JAMA Cardiol* 2021, **6**:437-447.
6. Demirci I, Haymana C, Candemir B, Meric C, Yuksel B, Eser M, Akin O, Akin S, Ersoz Gulcelik N, Sonmez A: **Triglyceride-glucose index levels in patients with congenital hypogonadotropic hypogonadism and the relationship with endothelial dysfunction and insulin resistance.** *Endokrynol Pol* 2021, **72**:232-237.
7. Ahn SH, Lee JH, Lee JW: **Inverse association between triglyceride glucose index and muscle mass in Korean adults: 2008-2011 KNHANES.** *Lipids Health Dis* 2020, **19**:243.
8. Jiang ZZ, Zhu JB, Shen HL, Zhao SS, Tang YY, Tang SQ, Liu XT, Jiang TA: **A High Triglyceride-Glucose Index Value Is Associated With an Increased Risk of Carotid Plaque Burden in Subjects With Prediabetes and New-Onset Type 2 Diabetes: A Real-World Study.** *Front Cardiovasc Med* 2022, **9**:832491.
9. Mirr M, Skrypnik D, Bogdanski P, Owecki M: **Newly proposed insulin resistance indexes called TyG-NC and TyG-NHtR show efficacy in diagnosing the metabolic syndrome.** *J Endocrinol Invest* 2021, **44**:2831-2843.
10. Kim MK, Ahn CW, Kang S, Nam JS, Kim KR, Park JS: **Relationship between the triglyceride glucose index and coronary artery calcification in Korean adults.** *Cardiovasc Diabetol* 2017, **16**:108.
11. Behnoush AH, Mousavi A, Ghondaghsaz E, Shojaei S, Cannavo A, Khalaji A: **The importance of assessing the triglyceride-glucose index (TyG) in patients with depression: A systematic review.** *Neurosci Biobehav Rev* 2024, **159**:105582.
12. Chen X, Liu D, He W, Hu H, Wang W: **Predictive performance of triglyceride glucose index (TyG index) to identify glucose status conversion: a 5-year longitudinal cohort study in Chinese pre-diabetes people.** *J Transl Med* 2023, **21**:624.
13. Sun Z, Liu J, Sun J, Xu Z, Liu W, Mao N, Chu T, Guo H, Che K, Xu X, et al: **Decreased Regional Spontaneous Brain Activity and Cognitive Dysfunction in Patients with Coronary Heart Disease: a Resting-state Functional MRI Study.** *Acad Radiol* 2023, **30**:1081-1091.
14. Lim J, Kim J, Koo SH, Kwon GC: **Comparison of triglyceride glucose index, and related parameters to predict insulin resistance in Korean

adults: An analysis of the 2007-2010 Korean National Health and Nutrition Examination Survey. *PLoS One* 2019, **14**:e0212963.
15. Sheng G, Lu S, Xie Q, Peng N, Kuang M, Zou Y: **The usefulness of obesity and lipid-related indices to predict the presence of Non-alcoholic fatty liver disease.** *Lipids Health Dis* 2021, **20**:134.
16. Cheng Y, Fang Z, Zhang X, Wen Y, Lu J, He S, Xu B: **Association between triglyceride glucose-body mass index and cardiovascular outcomes in patients undergoing percutaneous coronary intervention: a retrospective study.** *Cardiovasc Diabetol* 2023, **22**:75.
17. Liu L, Peng J, Wang N, Wu Z, Zhang Y, Cui H, Zang D, Lu F, Ma X, Yang J: **Comparison of seven surrogate insulin resistance indexes for prediction of incident coronary heart disease risk: a 10-year prospective cohort study.** *Front Endocrinol (Lausanne)* 2024, **15**:1290226.
18. Dang K, Wang X, Hu J, Zhang Y, Cheng L, Qi X, Liu L, Ming Z, Tao X, Li Y: **The association between triglyceride-glucose index and its combination with obesity indicators and cardiovascular disease: NHANES 2003-2018.** *Cardiovasc Diabetol* 2024, **23**:8.
19. Xuan W, Liu D, Zhong J, Luo H, Zhang X: **Impacts of Triglyceride Glucose-Waist to Height Ratio on Diabetes Incidence: A Secondary Analysis of A Population-Based Longitudinal Data.** *Front Endocrinol (Lausanne)* 2022, **13**:949831.
20. Wang Y, Zhuang Y, Lin C, Hong H, Chen F, Ke J: **The neutrophil-to-lymphocyte ratio is associated with coronary heart disease risk in adults: A population-based study.** *PLoS One* 2024, **19**:e0296838.
21. Yao Y, Wang B, Geng T, Chen J, Chen W, Li L: **The association between TyG and all-cause/non-cardiovascular mortality in general patients with type 2 diabetes mellitus is modified by age: results from the cohort study of NHANES 1999-2018.** *Cardiovasc Diabetol* 2024, **23**:43.
22. Abdalla SM, Yu S, Galea S: **Trends in Cardiovascular Disease Prevalence by Income Level in the United States.** *JAMA Netw Open* 2020, **3**:e2018150.
23. Che B, Zhong C, Zhang R, Pu L, Zhao T, Zhang Y, Han L: **Triglyceride-glucose index and triglyceride to high-density lipoprotein cholesterol ratio as potential cardiovascular disease risk factors: an analysis of UK biobank data.** *Cardiovasc Diabetol* 2023, **22**:34.
24. Zhao J, Fan H, Wang T, Yu B, Mao S, Wang X, Zhang W, Wang L, Zhang Y, Ren Z, Liang B: **TyG index is positively associated with risk of CHD and coronary atherosclerosis severity among NAFLD patients.** *Cardiovasc Diabetol* 2022, **21**:123.
25. da Silva A, Caldas APS, Hermsdorff HHM, Bersch-Ferreira AC, Torreglosa CR, Weber B, Bressan J: **Triglyceride-glucose index is associated with symptomatic coronary artery disease in patients in secondary care.** *Cardiovasc Diabetol* 2019, **18**:89.
26. Wang X, Xu W, Song Q, Zhao Z, Meng X, Xia C, Xie Y, Yang C, Jin P,

Wang F: **Association between the triglyceride-glucose index and severity of coronary artery disease.** *Cardiovasc Diabetol* 2022, **21**:168.

27. Wang J, Huang X, Fu C, Sheng Q, Liu P: **Association between triglyceride glucose index, coronary artery calcification and multivessel coronary disease in Chinese patients with acute coronary syndrome.** *Cardiovasc Diabetol* 2022, **21**:187.

28. Hu J, Cai X, Li N, Zhu Q, Wen W, Hong J, Zhang D, Yao X, Luo Q, Sun L: **Association Between Triglyceride Glucose Index-Waist Circumference and Risk of First Myocardial Infarction in Chinese Hypertensive Patients with Obstructive Sleep Apnoea: An Observational Cohort Study.** *Nat Sci Sleep* 2022, **14**:969-980.

29. Zhu Q, Chen Y, Cai X, Cai L, Hong J, Luo Q, Ren Y, Guo Y, Li N: **The non-linear relationship between triglyceride-glucose index and risk of chronic kidney disease in hypertensive patients with abnormal glucose metabolism: A cohort study.** *Front Med (Lausanne)* 2022, **9**:1018083.

30. Shi W, Xing L, Jing L, Tian Y, Yan H, Sun Q, Dai D, Shi L, Liu S: **Value of triglyceride-glucose index for the estimation of ischemic stroke risk: Insights from a general population.** *Nutr Metab Cardiovasc Dis* 2020, **30**:245-253.

31. Alizargar J, Bai CH, Hsieh NC, Wu SV: **Use of the triglyceride-glucose index (TyG) in cardiovascular disease patients.** *Cardiovasc Diabetol* 2020, **19**:8.

32. Iliodromiti S, Celis-Morales CA, Lyall DM, Anderson J, Gray SR, Mackay DF, Nelson SM, Welsh P, Pell JP, Gill JMR, Sattar N: **The impact of confounding on the associations of different adiposity measures with the incidence of cardiovascular disease: a cohort study of 296 535 adults of white European descent.** *Eur Heart J* 2018, **39**:1514-1520.

33. Miao H, Zhou Z, Yang S, Zhang Y: **The association of triglyceride-glucose index and related parameters with hypertension and cardiovascular risk: a cross-sectional study.** *Hypertens Res* 2024, **47**:877-886.

34. Chen J, Li M, Hao B, Cai Y, Li H, Zhou W, Song Y, Wang S, Liu H: **Waist to height ratio is associated with an increased risk of mortality in Chinese patients with heart failure with preserved ejection fraction.** *BMC Cardiovasc Disord* 2021, **21**:263.

35. Yang Q, Vijayakumar A, Kahn BB: **Metabolites as regulators of insulin sensitivity and metabolism.** *Nat Rev Mol Cell Biol* 2018, **19**:654-672.

36. Riehle C, Abel ED: **Insulin Signaling and Heart Failure.** *Circ Res* 2016, **118**:1151-1169.

37. Aparecida Silveira E, Vaseghi G, de Carvalho Santos AS, Kliemann N, Masoudkabir F, Noll M, Mohammadifard N, Sarrafzadegan N, de Oliveira C: **Visceral Obesity and Its Shared Role in Cancer and Cardiovascular**

Disease: A Scoping Review of the Pathophysiology and Pharmacological Treatments. *Int J Mol Sci* 2020, **21**.

38. Roy P, Orecchioni M, Ley K: **How the immune system shapes atherosclerosis: roles of innate and adaptive immunity.** *Nat Rev Immunol* 2022, **22**:251-265.

39. Hou P, Fang J, Liu Z, Shi Y, Agostini M, Bernassola F, Bove P, Candi E, Rovella V, Sica G, et al: **Macrophage polarization and metabolism in atherosclerosis.** *Cell Death Dis* 2023, **14**:691.

40. Gugliucci A: **Biomarkers of dysfunctional visceral fat.** *Adv Clin Chem* 2022, **109**:1-30.

9% Overall Similarity

Top sources found in the following databases:

- 8% Internet database
- 8% Publications database
- Crossref database
- Crossref Posted Content database

TOP SOURCES

The sources with the highest number of matches within the submission. Overlapping sources will not be displayed.

#	Source	Type	Similarity
1	cardiab.biomedcentral.com	Internet	<1%
2	researchsquare.com	Internet	<1%
3	ncbi.nlm.nih.gov	Internet	<1%
4	frontiersin.org	Internet	<1%
5	Anastasia V. Poznyak, Larisa Litvinova, Paolo Poggio, Vasily N. Sukhor...	Crossref	<1%
6	worldwidescience.org	Internet	<1%
7	tandfonline.com	Internet	<1%
8	dovepress.com	Internet	<1%
9	Zhen-zhen Jiang, Jian-bo Zhu, Hua-liang Shen, Shan-shan Zhao, Yun-yi...	Crossref	<1%

10	repository.cam.ac.uk<br>Internet	<1%
11	Alessandra da Silva, Ana Paula Silva Caldas, Helen Hermana Miranda ...<br>Crossref	<1%
12	Qida He, mengtong Sun, Hanqing Zhao, na Sun et al. "Ultra-processed f...<br>Crossref	<1%
13	Chen-Yuan Deng, Xin-Peng Ke, Xu-Guang Guo. "Investigating a novel s...<br>Crossref	<1%
14	Shuting Wang, Zhenzhou Shi, Hong Pan, Tiancai Yan, Ling Liu, Jiaheng ...<br>Crossref	<1%
15	Juan Ma, MoHan Wang, Peng Wu, Xueping Ma, Dapeng Chen, Shaobin ...<br>Crossref posted content	<1%
16	Yanjun Song, Kongyong Cui, Min Yang, Chenxi Song, Dong Yin, Qiuting ...<br>Crossref	<1%
17	Guowen Zhao, Sijia Shang, Na Tian, Xiaojiang Zhan et al. "Association ...<br>Crossref posted content	<1%
18	Yi Qin, Liping Xuan, Yujie Deng, Fei Wang, Bin Liu, Shujie Wang. "Triglyc...<br>Crossref	<1%
19	academic.oup.com<br>Internet	<1%
20	mdpi.com<br>Internet	<1%
21	Qurrat Ul Ain, Mehak Sarfraz, Gayuk Kalih Prasesti, Triwedya Indra Dew...<br>Crossref	<1%

22	assets.researchsquare.com<br>Internet	<1%
23	bmcgeriatr.biomedcentral.com<br>Internet	<1%
24	bmcpublichealth.biomedcentral.com<br>Internet	<1%
25	journals.plos.org<br>Internet	<1%
26	multimedia.elsevier.es<br>Internet	<1%
27	portailvasculaire.fr<br>Internet	<1%
28	Qing Zhu, Yuan Chen, Xintian Cai, Li Cai, Jing Hong, Qin Luo, Yingli Ren,...<br>Crossref	<1%
29	diabetesjournals.org<br>Internet	<1%
30	doaj.org<br>Internet	<1%
31	Rupeng Wang, Ce Chen, Guiyu Xu, Zening Jin. "Association of triglyceri...<br>Crossref	<1%
32	Xiaowan Li, Lan Cui, Hongyang Xu. "Association between systemic infl...<br>Crossref	<1%
33	archpublichealth.biomedcentral.com<br>Internet	<1%

34	Ningsheng Tian, Shuai Chen, Huawei Han, Jie Jin, Zhiwei Li. "Associati...<br>Crossref	<1%
35	Shaoyi Yan, Di Wang, Yongping Jia. "Comparison of insulin resistance-...<br>Crossref	<1%

Excluded from Similarity Report

- Bibliographic material
- Manually excluded text blocks

EXCLUDED TEXT BLOCKS

Clinical Medical College of Fujian Medical University

bmcmededuc.biomedcentral.com

These

journals.plos.org

AcknowledgementsWe thank the National Center for Health Statistics of the Cente...

bmcmedicine.biomedcentral.com

the NHANES data and

Huizhen Zheng, Ziwei Yin, Xi Luo, Yingli Zhou, Fei Zhang, Zhihua Guo. "Associations between systemic imm...

Authors' contributionsConceptualization, ZY and

Zahra Yari, Zahra Naser-Nakhaee, Elahe Karimi-Shahrbabak, Makan Cheraghpour et al. "Combination therap...

work was Sponsored by Fujian provincial health technology project (NO

Jieyu Li, Wansong Lin, Huijing Chen, Zhiping Xu, Yunbin Ye, Mingshui Chen. "Dual-target IL-12-containing na...

Writing-original draft, ZY

Zahra Yari, Zahra Naser-Nakhaee, Elahe Karimi-Shahrbabak, Makan Cheraghpour et al. "Combination therap...

Competing interestsThe authors declare that the research was conducted without ...

www.researchsquare.com

Consent for publicationNot applicable.Availability of data and materials

assets.researchsquare.com

www.cdc.gov/nchs/nhanes/(accessed on 15

assets.researchsquare.com

Ethics approval and consent to participateThe National Center for Health Statistics...

www.nature.com

OR: Odds ratio; CI: Confidence interval

www.researchsquare.com

CHD: Coronary heart disease

healthdocbox.com

TyG: Triglyceride glucose; TyG-WHtR: Triglyceride glucose-waist

www.frontiersin.org

waist to height ratio

Shaoyi Yan, Di Wang, Yongping Jia. "Comparison of insulin resistance-associated parameters in US adults: ...

Triglyceride glucose (TyG) index-related indicators

Wenting Xuan, Dixing Liu, Jiana Zhong, Huijin Luo, Xiuwei Zhang. "Impacts of Triglyceride Glucose-Waist to ...

to-height ratio

www.repository.cam.ac.uk

Data from

Ya Shao, Longti Li, Huiqin Zhong, Xiaojun Wang, Yu Hua, Xu Zhou. "Anticipated correlation between lean bod...

curve fitting and threshold effects analysis

Chen-Yuan Deng, Xin-Peng Ke, Xu-Guang Guo. "Investigating a novel surrogate indicator of adipose accumul...

and coronary heart disease were significantly

www.researchgate.net

smoking status, and

www.mdpi.com

and coronary heart disease

Keke Dang, Xuanyang Wang, Jinxia Hu, Yuntao Zhang, Licheng Cheng, Xiang Qi, Lin Liu, Zhu Ming, Xinmiao T...

was observed between TyG

Keke Dang, Xuanyang Wang, Jinxia Hu, Yuntao Zhang, Licheng Cheng, Xiang Qi, Lin Liu, Zhu Ming, Xinmiao T...

in the development of coronary heart disease

Valentin S Zhdanov, Nils H Sternby, Anatolii M Vikhert, Igor E Galakhov. "Development of atherosclerosis ov...

Insulin resistance

cardiab.biomedcentral.com

The TyGindex is a

Li-Chan Tao, Jia-ni Xu, Ting-ting Wang, Fei Hua, Jian-Jun Li. "Triglyceride-glucose index as a marker in cardi...

Triglyceride glucose-body mass index (TyG-BMI), Triglyceride glucose-waistcircu...

translational-medicine.biomedcentral.com

cross-sectional, population-based study

Huizhen Zheng, Ziwei Yin, Xi Luo, Yingli Zhou, Fei Zhang, Zhihua Guo. "Associations between systemic imm...

Figure 1).Figure 1: Flow chart

www.ncbi.nlm.nih.gov

coronary heart disease

Jiao Li, Zixian Dong, Hao Wu, Yue Liu, Yafang Chen, Si Li, Yufan Zhang, Xin Qi, Liping Wei. "The triglyceride-gl...

The association between

www.ncbi.nlm.nih.gov

correlation between

Juan Ma, MoHan Wang, Peng Wu, Xueping Ma, Dapeng Chen, Shaobin Jia, Ning Yan. "Predictive effect oftrig...

under 20 years of age

Huizhen Zheng, Ziwei Yin, Xi Luo, Yingli Zhou, Fei Zhang, Zhihua Guo. "Associations between systemic imm...

participants gave their written informedconsent to participate in the study

www.nature.com

low-density lipoproteins, and

www.cell.com

---

and coronary heart disease

www.ncbi.nlm.nih.gov

---

the risk of coronary heart diseaseincreased

www.ncbi.nlm.nih.gov

DiscussionTo the best of our knowledge, this is the first study to evaluate the asso...

bmcgeriatr.biomedcentral.com

---

in

cardiab.biomedcentral.com

---

coronary heart disease risk in the

Atlas of Atherosclerosis, 2000.

Table 5: Threshold effect.Outcome:Coronary heart disease riskModel IA straight-li...

journals.plos.org

Model IIFold points (K

www.ncbi.nlm.nih.gov

Log likelihood ratio tests0

www.ncbi.nlm.nih.gov

---

annual household income

www.tandfonline.com

---

the smooth curve fit between thevariables; the

Siying Lai, Lizi Zhang, Yang Luo, Zhongjia Gu et al. "A sonographic endometrial thickness <7 mm in women ...

---

and coronary heart disease risk was

www.ncbi.nlm.nih.gov

Smooth curve fittingrevealed a U-shaped

www.researchsquare.com

coronary heart disease

www.ncbi.nlm.nih.gov

Fully adjusted

www.ncbi.nlm.nih.gov

annual household income

www.tandfonline.com

Table 4: Multivariate analysis of associations between various variables and coron...

journals.plos.org

genderFemaleMaleRaceMexican AmericanOther HispanicNon-Hispanic WhiteNon-...

Xiaoyi Qi, Shijia Wang, Qianwen Huang, Xiongbiao Chen, Liangxian Qiu, Kunfu Ouyang, Yanjun Chen. "The as...

Crude modelMinimally adjusted model Fully adjusted model

www.frontiersin.org

Crude Model: No covariates were adjusted.Minimally adjusted model: Age

Sheng Wan, Xiaobo Zhao, Jindan Pei, Zhimin Han, Ronghua Che, Shi Qin, Xiaolin Hua. "Association of age at ...

annual household income

www.nature.com

Table 3: Association between

cardiab.biomedcentral.com

Mean±SD for continuous variables: the P value was calculated by the weighted line...

assets.researchsquare.com

Cholesterol (mmol/L)4

bmccardiovascdisord.biomedcentral.com

Education level (%)0.001Less than 9th grade

www.researchsquare.com

0.001Mexican American

www.ncbi.nlm.nih.gov

Table 2: Characteristics of the study population based on coronary heart disease....

journals.plos.org

Cholesterol (mmol/L)4

Jie Huang, Jiaheng Han, Rigbat Rozi, Bensheng Fu, Zhengcao Lu, Jiang Liu, Yu Ding. "Association between li...

Mean ± SD for continuous variables: the P value was calculated by the weighted lin...

assets.researchsquare.com

Coronary heart disease (%)<0.001Yes

Huizhen Zheng, Ziwei Yin, Xi Luo, Yingli Zhou, Fei Zhang, Zhihua Guo. "Associations between systemic imm...

Education level (%)<0.001Less than 9th grade5

Siyu Ma, Jie Zhang, Cheng Xu, Min Da, Yang Xu, Yong Chen, Xuming Mo. "Increased Serum Levels of Cadmi...

Race (%)<0.001Mexican American

lipidworld.biomedcentral.com

gender (%)<0.001Male42

bmcpublichealth.biomedcentral.com

Table 1: Characteristics of the study population based on

journals.plos.org

Tertiles 1Tertiles 2Tertiles 3P value

Prakash Chand Negi, Chander Kant Sharma, Rahul Nihjawan, Rajesh Sharma, Sanjeev Asotra. "Role of omeg...

P<0.05). Participants in the

assets.researchsquare.com

Statistical significance was set at P＜0.05.ResultsBaseline Characteristics of Parti...

josr-online.biomedcentral.com

R Studio (version 4

www.frontiersin.org

the study

bmcmusculoskeletdisord.biomedcentral.com

multivariate logistic regressionanalysis was used to determine the odds ratio (OR) ...

www.researchsquare.com

in

www.mdpi.com

the NHANES website: www.cdc.gov/nchs/nhanes/.Statistical analysisAll statistica...

www.frontiersin.org

based on consuming more than 12 drinks per year

lipidworld.biomedcentral.com

Smoking status wasdetermined by

www.researchsquare.com

Coronary heart disease

www.ncbi.nlm.nih.gov

coronary heart disease

www.ncbi.nlm.nih.gov

WHtR=waist circumference/height;TyG-WHtR=TyG×WHtR

Yuansong Zhuang, Liliang Qiu, Dongjian Han, Zhentao Qiao et al. "The association between triglyceride-gluc...

The following

www.frontiersin.org

triglyceride and fasting blood glucose levels to

Lin Na, Wenjing Cui, Xinqi Li, Jing Chang, Xin Xue. "Association between the triglyceride–glucose index and l...

The National Center for Health Statistics Research EthicsReview Board

www.mdpi.com

atherosclerotic plaques form in the coronaryarteries

Wei Yang, Xuguang Li, Xuemei Li, Baoping Hu, Shilin Xu, Hengxun Zhang, Yuhe Wang, Tianbo Jin, Yongjun H...

was examined

Shuting Wang, Zhenzhou Shi, Hong Pan, Tiancai Yan, Ling Liu, Jiaheng Xu, Wei Wang, Tong Zhang. "Triglyce...

cross-sectional study

www.ncbi.nlm.nih.gov

Mexican American, Other Hispanic, Non-Hispanic White, Non-Hispanic Black

www.frontiersin.org

They

www.ncbi.nlm.nih.gov

based on gender, age,smoking status

www.ncbi.nlm.nih.gov

Non-HispanicWhite

assets.researchsquare.com

for age, gender, race, annual householdincome, educational level, smoking status, ...

www.tandfonline.com

coronary heart disease (P<0.05). Patients with coronary heart disease

www.scienceopen.com

tended to be older, male

bmcpublichealth.biomedcentral.com

The findings of the multivariate regression analysis

Jiao Li, Zixian Dong, Hao Wu, Yue Liu, Yafang Chen, Si Li, Yufan Zhang, Xin Qi, Liping Wei. "The triglyceride-gl...

and coronary heart disease

www.ncbi.nlm.nih.gov

the crude (OR=2

www.ncbi.nlm.nih.gov

higher risk of coronary heart disease than those in Tertile 1

www.ncbi.nlm.nih.gov

with P values

docksci.com

cardiovascular riskfactors

cardiab.biomedcentral.com

an independent

Jiao Li, Zixian Dong, Hao Wu, Yue Liu, Yafang Chen, Si Li, Yufan Zhang, Xin Qi, Liping Wei. "The triglyceride-gl...

The TyG

www.researchsquare.com

The TyG index

Jiao Li, Zixian Dong, Hao Wu, Yue Liu, Yafang Chen, Si Li, Yufan Zhang, Xin Qi, Liping Wei. "The triglyceride-gl...

than

Kyungchul Song, Goeun Park, Hye Sun Lee, Myeongseob Lee et al. "Comparison of the Triglyceride Glucose ...

aremore accurate predictors of

Rujikorn Rattanatham, Jitbanjong Tangpong, Moragot Chatatikun, Dali Sun et al. "Assessment of eight insuli...

Insulin resistance

Kyungchul Song, Goeun Park, Hye Sun Lee, Myeongseob Lee et al. "Comparison of the Triglyceride Glucose ...

in the United States to

lipidworld.biomedcentral.com

NHANES study

Hao Yang, Hong Lin, Xiaorong Liu, Haoran Liu, Ting Chen, Zhaohui Jin. "Association between dietary fiber int...

Continuous data are represented

www.cambridge.org

as

www.frontiersin.org

the minimally adjusted model was adjusted for age, gender,and race

www.frontiersin.org

A threshold

www.frontiersin.org

of the participants.The clinical features of the

Sanghoon Kim, Ji-Won Lee, Yaeji Lee, Youhyun Song, John A Linton. "Association between triglyceride-gluco...

was converted from continuous to categorical

www.frontiersin.org

in both males (OR=1

Connie W. Tsao, Aaron W. Aday, Zaid I. Almarzooq, Alvaro Alonso et al. "Heart Disease and Stroke Statistics...

suggesting that these factors didnot

assets.researchsquare.com

had a U-shaped

Connie W. Tsao, Aaron W. Aday, Zaid I. Almarzooq, Cheryl A.M. Anderson et al. "Heart Disease and Stroke St...

were associated with an increased riskof coronary heart disease

www.researchgate.net

association between TyG

cardiab.biomedcentral.com

increased risk of adverse cardiovascular events

www.researchsquare.com

the association between obesity and

cardiab.biomedcentral.com

coronary heart disease

www.ncbi.nlm.nih.gov

indicated a

www.ncbi.nlm.nih.gov

Race was categorized as

cardiab.biomedcentral.com

The Centers for Disease Control and Prevention

www.researchsquare.com

models. The crude modelwas not adjusted for

www.researchsquare.com

variableswere evaluated

cardiab.biomedcentral.com
